# Boosting the thermoelectric performance of p-type heavily Cu-doped polycrystalline SnSe *via* inducing intensive crystal imperfections and defect phonon scattering†
†Electronic supplementary information (ESI) available: Fig. S1–S8 and Table S1. See DOI: 10.1039/c8sc02397b


**DOI:** 10.1039/c8sc02397b

**Published:** 2018-07-30

**Authors:** Xiaolei Shi, Kun Zheng, Min Hong, Weidi Liu, Raza Moshwan, Yuan Wang, Xianlin Qu, Zhi-Gang Chen, Jin Zou

**Affiliations:** a Materials Engineering , The University of Queensland , Brisbane , QLD 4072 , Australia . Email: j.zou@uq.edu.au; b Institute of Microstructure and Properties of Advanced Materials , Beijing University of Technology , Beijing 100022 , China; c Centre for Future Materials , University of Southern Queensland , Springfield , QLD 4300 , Australia . Email: zhigang.chen@usq.edu.au; d Centre for Microscopy and Microanalysis , The University of Queensland , Brisbane , QLD 4072 , Australia

## Abstract

In this study, we, for the first time, report a high Cu solubility of 11.8% in single crystal SnSe microbelts synthesized *via* a facile solvothermal route. The pellets sintered from these heavily Cu-doped microbelts show a high power factor of 5.57 μW cm^–1^ K^–2^ and low thermal conductivity of 0.32 W m^–1^ K^–1^ at 823 K, contributing to a high peak *ZT* of ∼1.41. Through a combination of detailed structural and chemical characterizations, we found that with increasing the Cu doping level, the morphology of the synthesized Sn_1–*x*_Cu_*x*_Se (*x* is from 0 to 0.118) transfers from rectangular microplate to microbelt. The high electrical transport performance comes from the obtained Cu^+^ doped state, and the intensive crystal imperfections such as dislocations, lattice distortions, and strains, play key roles in keeping low thermal conductivity. This study fills in the gaps of the existing knowledge concerning the doping mechanisms of Cu in SnSe systems, and provides a new strategy to achieve high thermoelectric performance in SnSe-based thermoelectric materials.

## Abstract 2

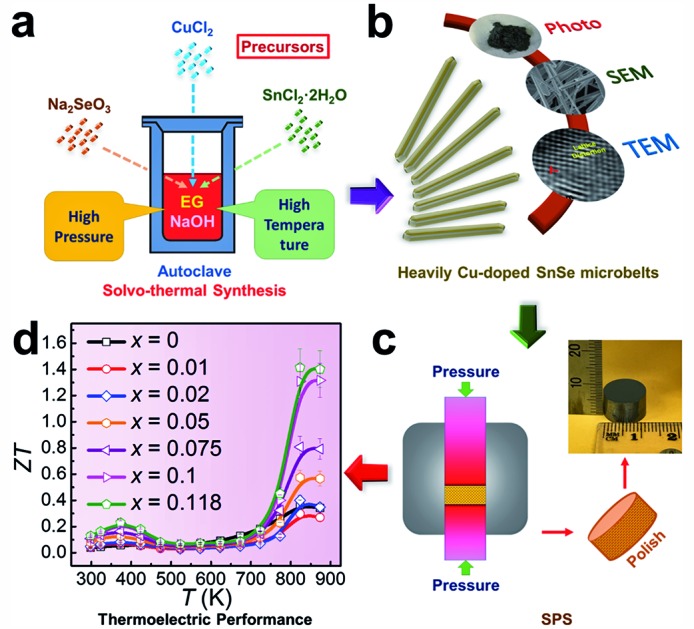
In this study, we, for the first time, report a high Cu solubility of 11.8% in single crystal SnSe microbelts synthesized *via* a facile solvothermal route.

## Introduction

With the capability of directly converting between heat and electricity, thermoelectric materials provide a promising alternative energy supplement in applications by collecting the waste-heat and assisting in finding new energy solutions.^1^,^2^ To evaluate the converting efficiency, the unitless figure of merit *ZT* is defined as *ZT* = *S*^2^*σT*/*κ* and *κ* = *κ*_e_ + *κ*_l_, where *σ*, *S*, *κ*, *κ*_l_, *κ*_e_, and *T* are the electrical conductivity, the Seebeck coefficient, the thermal conductivity, the lattice thermal conductivity, the electrical thermal conductivity, and the absolute temperature,^3^–^5^ respectively. A high *ZT* needs a low *κ* and a high power factor (*S*^2^*σ*). Since *S*, *σ* and *κ*_e_ are strongly coupled through the carrier concentration (*n*), achieving high *ZT* values has been historically difficult. It is therefore essential to explore favourable electrical transport properties to strengthen the energy conversion efficiency, and to realize a low thermal transport speed to relieve the heat loss at the same time. To achieve this goal, with a narrow band-gap of ∼0.9 eV,^2^,^6^,^7^ tin selenide (SnSe) has received great attention for applications in low-cost thermoelectrics.^8^–^11^ A remarkably high peak *ZT* of ∼2.6 has been reported along the *b*-axis of p-type SnSe crystals,^8^ where the performance benefits from the crystals’ reasonable *σ* and low *κ* values at 923 K.^12^ However, as they suffer from potentially high production costs and poor mechanical properties, SnSe crystals are difficult to use in thermoelectric devices, and their critical crystal-growth techniques have considerable limitations for industrial scale-up.^13^ Meanwhile, there is strong controversy over the high *ZT* of SnSe crystals due to the fact that the *κ* values determined in these crystals are not their intrinsic values,^14^,^15^ and the reinvestigation of single crystals has demonstrated much higher *κ* values.^15^ To overcome these challenges, polycrystalline SnSe has been considered as an alternative approach.^16^ However, due to the low *σ* values derived from low *n* (<10^18^ cm^–3^), the *ZT* values (<0.3) have been found to be undesirable for un-doped polycrystalline SnSe.^8^ As indicated from previous calculations,^17^,^18^ the optimised *n* value of p-type SnSe is ∼3 × 10^19^ cm^–3^ to reach an enhanced *ZT* value, so that there is a great potential to enhance these values through effective engineering.

Doping and/or alloying have been widely used for tuning *n* to achieve desired *σ* values.^19^,^20^ Various elements, such as alkali metals (Na and K),^21^–^28^ I-B group metals (Cu and Ag),^29^–^36^ and halogens (Cl, Br and I),^37^–^41^ have been used as dopants in either p-type or n-type SnSe.^16^ As a typical I-B group metal and its abundant availability in earth, Cu, each atom having one valence electron (similar to alkali metals), becomes a good candidate to for tuning *n*,^29^ and in turn for improving *σ*.^31^ However, the fundamental mechanisms, such as the Cu doping limit and its valence state in SnSe, are still unclear. Recent studies have shown that to achieve homogeneous Cu doping in SnSe is a challenge,^29^ and the secondary phase (such as Cu_2_Se) generated during the synthesis is difficult to remove from the system *via* the post-melting route.^29^ Furthermore, there is no direct structural evidence to demonstrate the doping behaviours of Cu in SnSe crystals. Therefore, urgent attention is needed to clarify these fundamentals *via* critical structural and chemical characterizations, which will illustrate the doping behaviours, and effectively improve *σ* to benefit the energy conversion efficiency.

**Fig. 1:**
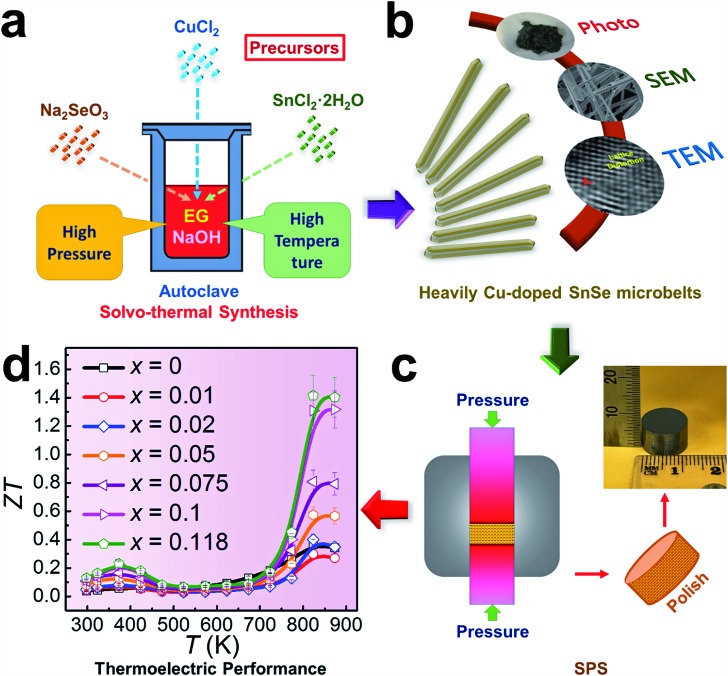
Illustrations of heavily Cu-doped SnSe (Sn_1–*x*_Cu_*x*_Se): (a) fabrication process; (b) characterization techniques used; (c) sintering process and (d) obtained *ZT* values.

To explore these fundamental mechanisms and achieve a high thermoelectric performance at both low and high temperatures, in this study we fabricated Cu-doped SnSe microbelts *via* a simple solvothermal method as illustrated in Fig. 1(a), from which a high doping limit of Cu (11.8%) in SnSe microbelts was achieved for the first time. The secondary phase (Cu_2_Se) in the synthesized products can be found when excessive Cu is doped in SnSe, but this was effectively removed through sonic separation and centrifuging after the solvothermal synthesis. Through detailed structural characterization as illustrated in Fig. 1(b), it was found that with increasing the Cu doping level, the morphology of Sn_1–*x*_Cu_*x*_Se (*x* is from 0 to 0.118) can be tuned from rectangular plates to microbelts. Both Cu^+^ and Cu^2+^ valence states were confirmed in the synthesized Sn_1–*x*_Cu_*x*_Se *via* XPS analysis. The observed lattice distortion plays a dominant role in keeping the heavily doped SnSe microbelts in the orthorhombic structure. After being sintered into pellets as illustrated in Fig. 1(c), the comprehensive thermoelectric properties, such as carrier mobility (*μ*), *n*, *σ*, *S*, *S*^2^*σ*, and *κ*, were measured and calculated, which led to a high *ZT* of ∼1.41 at 823 K when *x* = 0.118, as shown in Fig. 1(d), indicating that our heavily Cu-doped SnSe has full potential for applications in high temperature thermoelectric devices.

## Results and discussion

To understand the extraordinary thermoelectric performance found in our heavily Cu-doped SnSe, we first investigated the solubility of Cu in SnSe *via* X-ray diffraction (XRD) analysis and electron probe micro-analysis (EPMA), and then studied the valence state of Cu in SnSe *via* X-ray photoelectron spectroscopy (XPS). Detailed characterizations by scanning electron microscopy (SEM), high resolution transmission electron microscopy (HR-TEM), spherical aberration corrected scanning transmission electron microscopy (Cs-STEM) with high-angle annular dark-field (HAADF) imaging and energy dispersive spectroscopy (EDS) are presented and discussed to explain the fundamental reasons for the obtained high thermoelectric performance.

In this study, we use Na_2_SeO_3_ as the Se source, SnCl_2_·2H_2_O as the Sn source, and CuCl_2_ as the Cu doping source. To study the solubility of Cu in SnSe, we define the molar percentage *r* of CuCl_2_ in the total amount of CuCl_2_ and SnCl_2_·2H_2_O. The selected *r* values in this study were 0% (no CuCl_2_ added), 1%, 2%, 5%, 7.5%, 10%, 20%, and 30%, respectively. Through detailed EPMA studies, the Cu doping level (defined as *x* for Sn_1–*x*_Cu_*x*_Se) from different *r* values was found as 0%, 1%, 2%, 5%, 7.5%, 10%, 11.8%, and 11.8%, respectively, indicating that the solubility of Cu in the SnSe system is 11.8%. In the cases of *r* = 20% and 30%, an obvious secondary phase of Cu_2_Se can be identified when the doping concentration is beyond the solubility (11.8%) of Cu in the SnSe system. However, the secondary phase can be effectively removed through ultrasonic separation and centrifuging techniques after the solvothermal synthesis; a detailed discussion is shown in the ESI, Fig. S1(a–c).† Therefore, the obtained final synthesized products with *r* = 20% and 30% are almost single-phase Sn_0.882_Cu_0.118_Se microbelts.

**Fig. 2:**
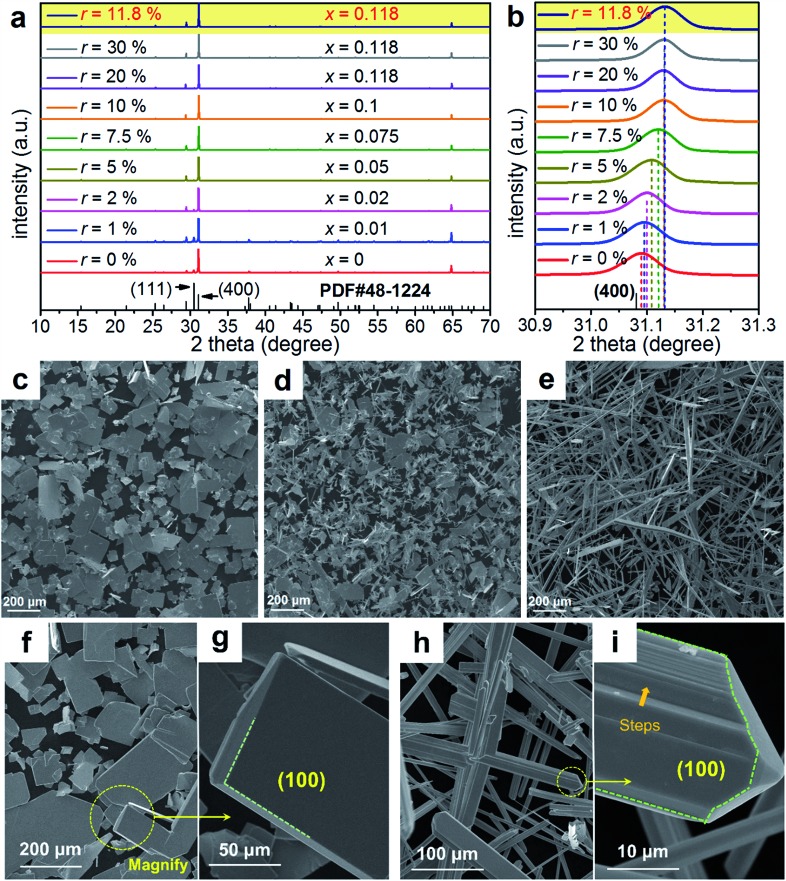
(a) XRD patterns of synthesized products with different *r* and *x* values. (b) Magnified XRD patterns to see the peak deviation at 400*. SEM images of synthesized products for (c) *x* = 0, (d) *x* = 0.05, and (e) *x* = 0.118, to see the morphology variation. (f) Magnified SEM image of synthesized products for *x* = 0. (g) Magnified SEM image of the circled area in (f) to show the (100) surface. (h) Magnified SEM image of synthesized products for *x* = 0.118. (i) Magnified SEM image of the circled area in (h) to show the (100) surface.

Investigating the structural characteristics of our synthesized products, Fig. 2(a) shows their XRD patterns. All diffraction peaks for all products can be exclusively indexed as the orthorhombic-structured SnSe, and a *Pnma* space group (Standard Identification Card, JCPDS 48-1224). As can be seen in Fig. 2(a), the strongest peak is the 400* peak for all products, suggesting that all products should possess significant {100} surfaces. Because the 400* peak is much more significant than the other peaks, it is hard to see most of the peaks in detail. To solve this problem, we magnified one of our XRD patterns (*r* = 20%) as shown in Fig. S1(c),† from which all peaks can be exclusively indexed as the orthorhombic-structured SnSe, and no secondary phase can be found. Fig. 2(b) shows detailed 400* diffraction peaks for different *r* values, indicating that for *r* > 0%, all 400* peaks deviate from the standard value at 2*θ* = 31.081°. Even for *r* = 0%, the slightly right-shifted 400* peak indicates the Sn vacancies exist in the SnSe structure.^10^ Our extensive EPMA studies found that the true atomic ratio of Sn : Se is ∼0.996 : 1. With an increase of the Cu doping level, the 400* peaks shift towards a higher 2*θ*, indicating that Cu atoms are incorporated into the SnSe lattice. Because the size of Cu ions is smaller than Sn ions, the Cu-doping leads to a decrease of the lattice parameter *a*.^42^ However, for *r* > ∼10%, no further observable shift of the 400* peak suggests that the doping limit of Cu in SnSe is reached, agreeing with the EMPA results, which is a surprising value. To doubly confirm this, we also synthesized products with *r* = 11.8%, as shown in the yellow-highlighted regions in both Fig. 2(a) and (b). It is clear to see that the peak shift from *r* = 11.8% is same as that from *r* = 20% and *r* = 30%, indicating that the solubility of Cu in the SnSe structure is 11.8%. A detailed discussion about the variations of the calculated lattice parameters (*a*, *b*, and *c*) and unit cell volume can be seen in Fig. S2 in the ESI.†

Cu doping has been reported to contribute to a morphology and/or facet change for many materials during their single crystal growth *via* various solution methods.^43^–^45^ For the case of single-crystal SnSe synthesized *via* our solvothermal route, morphological evolution in Cu-doped SnSe was also observed. Fig. 2(c–e) show typical SEM images of the synthesized products for *x* = 0, 0.05, and 0.118 (*r* = 20%), respectively. For *x* = 0, as shown in Fig. 2(c), the synthesized products have a typical rectangular plate-like morphology, and their lateral dimensions vary between 30 and 200 μm, similar to the reported morphology.^10^,^46^ Interestingly, with increasing *x*, the morphology of SnSe gradually transfers from rectangular plate-like into long belt-like morphology. More evidences of the morphology transition are shown in Fig. S3(a–f) in the ESI.† To determine the preferred facets for different *x* values, detailed SEM investigations were performed. Fig. 2(f) shows a SEM image of the synthesized SnSe plates with *x* = 0, from which the circled area is magnified as shown in Fig. 2(g), in which the (100) surface is labelled. It is of interest to note that, compared with other surfaces, the SnSe microplates possess significant {100} surfaces, which explain why 400* is the strongest peak. To illustrate the potential surfaces of our SnSe microplates, we simulated the single crystal microplate of SnSe using software (WinXMorph),^47^ and the corresponding crystal model is shown in Fig. S4(a) of the ESI.† On the other hand, Fig. 2(h) shows the SEM image taken from a typical Sn_0.882_Cu_0.118_Se microbelt, from which the circled area is also magnified as shown in Fig. 2(i) with the labelled (100) surface. {100} are still the most significant surfaces on the microbelts. Besides, Fig. 2(i) shows many surface steps parallel to the axial direction of the belt, which is likely to be caused by the irregular stacking of Sn–Se thinner belts. To illustrate the facets of our heavily Cu-doped SnSe, we also simulates the single-crystal microbelts using software (WinXMorph),^47^ and the corresponding crystal model is shown in Fig. S4(b) of the ESI.†

**Fig. 3:**
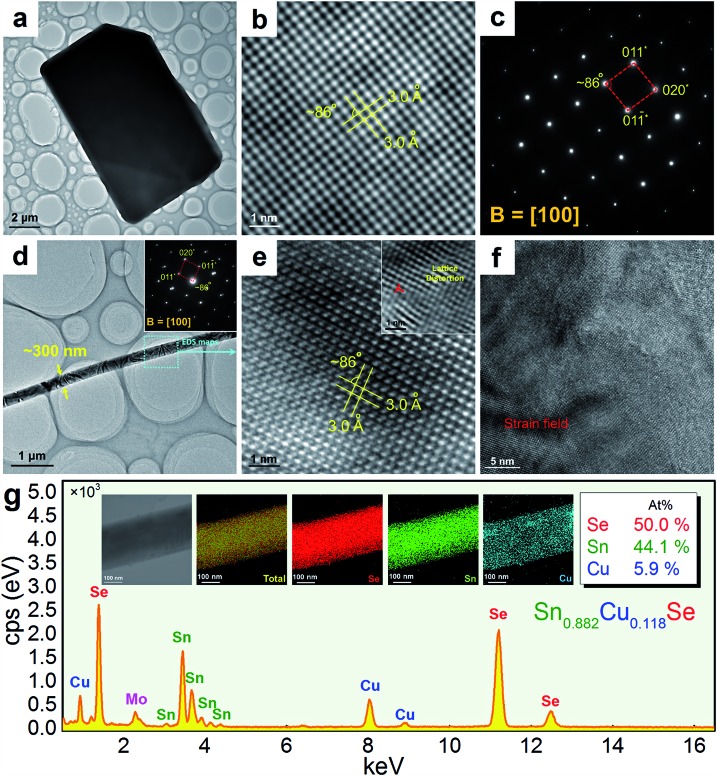
(a) TEM image of a typical SnSe microplate for *x* = 0, corresponding (b) HRTEM image and (c) SAED pattern taken from the plate shown in (a). (d) TEM image of a section of a typical SnSe microbelt for *x* = 0.118, inset showing the corresponding SAED pattern, (e) corresponding HRTEM image with a dislocation shown in the inset. (f) TEM image showing significant strain contrast. (g) EDS map and spot analysis taken from a typical SnSe microbelt for *x* = 0.118.

Fig. 3(a) shows a TEM image taken from a typical SnSe microplate, in which the electron beam is parallel to the normal direction of the plate. Fig. 3(b) and (c) are the HRTEM image and selected area electron diffraction (SAED) pattern taken from the thin corner area of the plate, and show that the plate has the orthorhombic structure and has a {100} surface. Fig. 3(d) is a TEM image taken from a section of a typical Sn_0.882_Cu_0.118_Se microbelt with a width of ∼300 nm. The inset is the SAED pattern taken along the *d* zone-axis, showing that the axial direction of the belt is parallel to the direction. Fig. 3(e) is the corresponding HRTEM image, showing the typical orthorhombic structure. Dislocations are often found through our HRTEM investigations, and an example is shown in the inset in Fig. 3(e). Fig. 3(f) is an HRTEM image taken from a relatively larger area in a belt, and shows a significant strain contrast. Such a strain contrast could be caused by the local non-uniformity of Cu doping and a possible mixture of Cu^+^ and Cu^2+^. To confirm this, energy dispersive spectroscopy (EDS) mapping was performed. We used a Mo grid rather than a Cu grid to avoid Cu impact from the grid. Fig. 3(g) shows respective the EDS maps for Se, Sn, and Cu, and overlapped images from a typical microbelt. All of the elements are well distributed, indicating the successful doping of Cu in the SnSe system. The local non-uniformity of Cu can also be seen. Besides, extensive EDS measurements are used to analyse the Cu concentration, and an example is shown in Fig. 3(g), which agrees with our EPMA analysis.

**Fig. 4:**
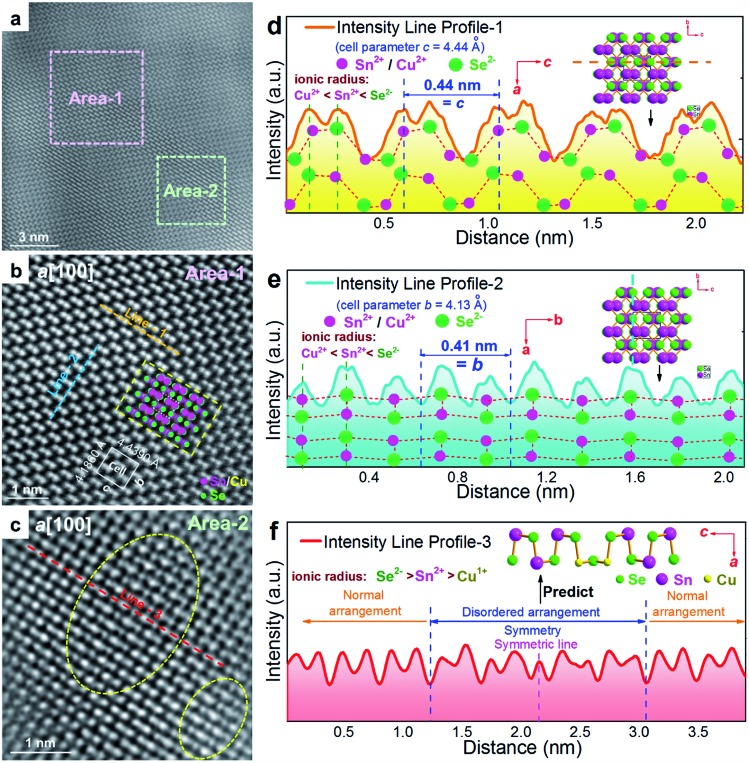
(a) STEM HAADF image of microbelt (*x* = 0.118) viewed along the *a*-direction with strain fields. Area-1 was taken from a normal area and Area-2 was taken from an area across the strain field. HR-STEM HAADF images of (b) Area-1 and (c) Area-2. The overlays in (b) show cell parameters, axes, and Sn/Cu atoms in purple and Se atoms in green. The yellow dashed circles in (c) show the areas with a disordered arrangement of atoms. (d) Intensity line profile-1 with illustrated crystal structure viewed along the *b*-axis, (e) intensity line profile-2 taken from (b) with illustrated crystal structure viewed along the *c*-axis, and (f) intensity line profile-3 taken from (c).

To understand the detailed structural characteristics of the Sn(Cu)/Se slabs stack, Cs-corrected STEM-HAADF investigations were performed. Fig. 4(a) is a STEM-HAADF image taken from a typical Sn_0.882_Cu_0.118_Se microbelt viewed along the *a*-axis, which also shows non-uniform contrast and varied structural patterns, suggesting the local elemental variation. This explains the strain contrast observed in Fig. 3(f). In fact, such local compositional variation and dislocations cause lattice distortions, which in turn enhance the phonon scatterings. Fig. 4(b) and (c) show HR-STEM HAADF images of Area-1 and Area-2 indicated in Fig. 4(a), respectively. For Area-1, the overlays in Fig. 4(b) show lattice parameters, axes, and Sn/Cu atoms in purple and Se atoms in green (shown in the dashed rectangle). The dotted white rectangle in the centre of the overlay indicates the projected unit cell, and the theoretical values of *c* and *b* are 4.439 Å and 4.186 Å, respectively.^48^,^49^ The clear atomic structure of SnSe with no atom disarrangement was observed. Fig. 4(d) and (e) are the intensity line profile-1 (dashed orange line) taken along the *c*-axis and profile-2 (dashed blue line) taken along the *b*-axis in Fig. 4(b), respectively. As can be seen, the measured cell parameter for *c* was ∼0.44 nm, which is close to the calculated value (4.44 Å). Similarly, the measured cell parameter of *b* in Fig. 4(e) is ∼0.41 nm, which also is close to the calculated value (4.13 Å). All of these evidences demonstrate the nature of the orthorhombic structure of SnSe. Considering the slight difference between peak intensities shown in Fig. 4(d) and (e), it is predicted that Cu^2+^ substitutes the position of Sn^2+^, resulting in weakened peaks. For Area-2, the yellow dashed circles in Fig. 4(c) show the areas with a disordered arrangement of atoms. Fig. 4(f) is an intensity line profile-3 taken from Fig. 4(c) (dashed red line) along the *c*-axis, from which the measured disordered arrangement of atoms possesses a symmetry line, indicating the potential existence of Cu^+^ illustrated by the inserted crystal structure in Fig. 4(f).

**Fig. 5:**
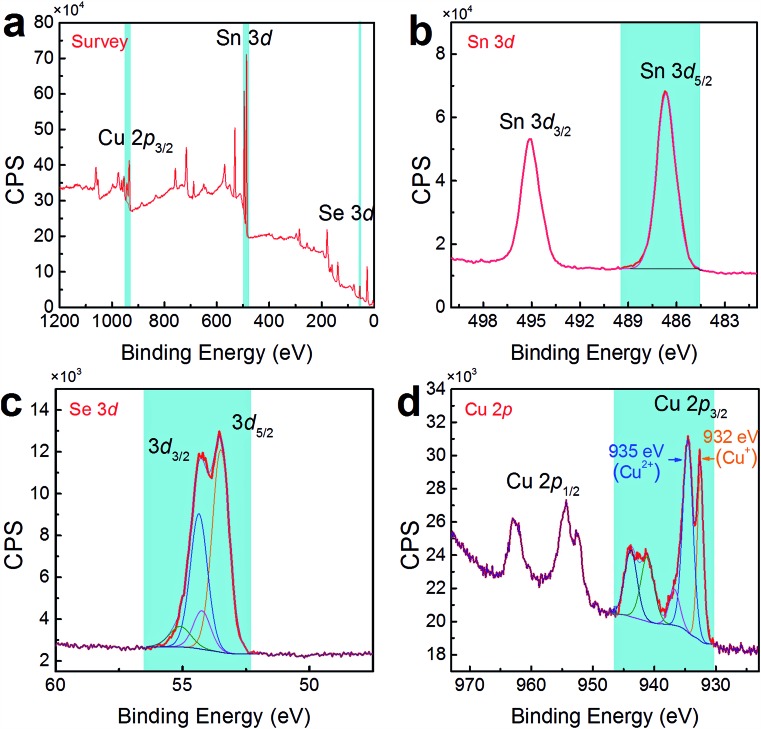
(a) Survey scan of XPS spectra for synthesized products with *x* = 0.118. High-resolution scans of XPS spectra for (b) Sn 3d, (c) Se 3d, and (d) Cu 2p.

To confirm the co-existence of Cu^+^ and Cu^2+^ in our Cu-doped SnSe, XPS analysis was performed. Fig. 5(a) shows the survey scan for synthesized Sn_0.882_Cu_0.118_Se microbelts, indicating the presence of Sn 3d, Se 3d, and Cu 2p energy states, without any energy states of other elements except for O and C. To analyse the detailed information of Sn, Se, and Cu, Fig. 5(b–d) respectively show high-resolution scans of XPS spectra for Sn 3d, Se 3d, and Cu 2p, from which both Sn and Se atoms present single valence states. For Sn, the peaks corresponding to Sn 3d_3/2_ and Sn 3d_5/2_ are singlets, and no accessorial binding energy peaks can be found, indicating the divalent characteristic of the Sn ions. For Se, a binding energy peak at 53.7 eV corresponds to Se 3d.^46^ For Cu, as shown in Fig. 5(d), strong peaks corresponding to Cu 2p_3/2_ were observed at ∼933 eV, indicating the successful doping in SnSe. Interestingly, there were two valence states for the Cu ions (Cu^+^ for the peak at 932 eV and Cu^2+^ for the peak at 935 eV) in SnSe, which is a new finding in the doping behaviour of Cu. The quantified at% of Cu agreed with the proposed 11.8% of Cu.

**Fig. 6:**
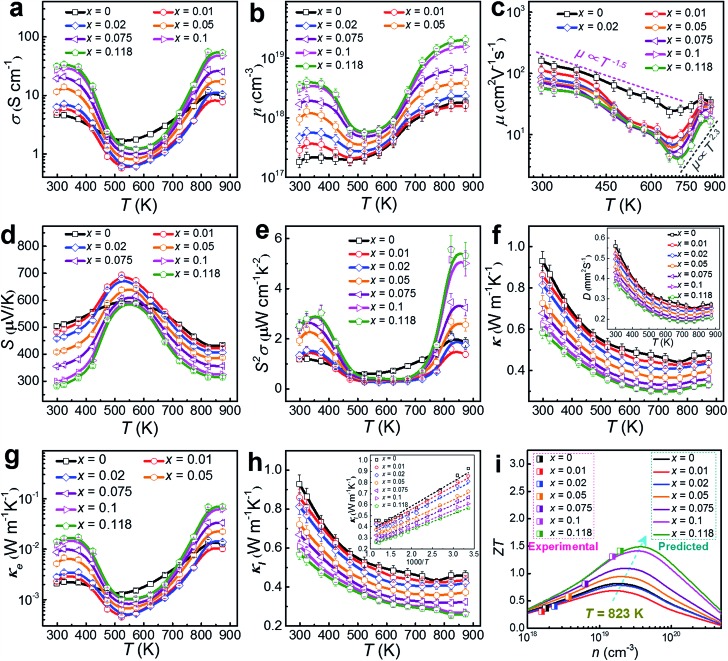
Plots of measured properties of (a) *T*-dependent *σ*; (b) *T*-dependent *n*; (c) *T*-dependent *μ*; (d) *T*-dependent *S*; (e) *T*-dependent *S*^2^*σ*; (f) *T*-dependent *κ* with inset of *T*-dependent *D*; (g) *T*-dependent *κ*_e_, (h) *T*-dependent *κ*_l_ with inset of 1000/*T*-dependent *κ*_l_ and (i) comparison of experimental *ZT*s with predicted plots for our Cu-doped SnSe pellets.

To understand the thermoelectric properties of our Cu-doped SnSe microbelts, we sintered as-synthesized products (with *x* = 0, 0.01, 0.02, 0.05, 0.075, 0.1 and 0.118, respectively) into pellets, and cut the pellets into rectangular chips to measure and calculate the key properties (*σ*, *S*, *S*^2^*σ* and *κ*) between 300 and 873 K. Considering that all properties except *S* measured along the ⊥ directions (perpendicular to the sintering pressure) are higher than those measured along the ∥ directions (parallel to the sintering pressure) due to the anisotropy (shown in Fig. S5 in the ESI†),^8^,^10^,^50^ we chose the ⊥ direction as the main measured direction in the following discussions. Fig. 6(a) shows the measured temperature-dependent *σ* parameters for pellets with different *x* values. After doping with Cu, the *σ* values were greatly enhanced at low temperature (from 300 to 450 K) and high temperature (above 773 K) when *x* = 0.118. As can be seen, two regions for *σ* exist. From 323 to 573 K (the first region), a typical metallic transport behaviour can be observed. After being heavily doped with Cu, the metal cations (especially Cu^+^) increased. In this situation, with increasing the temperature, the vibration of the metal cations becomes more intensive than their un-doped counterparts, which severely impede the carrier transport, resulting in a drastic drop in *σ*.^8^ From 573 to 873 K (the second region), typical thermally activated semiconducting behaviour derived from the thermal excitation of the carriers is seen, which is similar to the case of single crystals.^8^ Besides, the strong bipolar effect,^51^ arising between 500 and 600 K, can produce additional holes, leading to a rapid *n* increase, and in turn increasing *σ*.^18^ These results indicate that the doped Cu (mainly Cu^+^) can significantly improve the *σ* of pure SnSe at high temperature by strengthening the thermal excitation of the carriers, even though it results in a slight reduction of *σ* at medium temperature, which is why the pure SnSe sample outperforms most of the Cu-doped samples in this temperature range.

As discussed above, the greatly enhanced *σ* after being heavily doped with Cu should come from the *n* enhancement in the Cu-doped SnSe, as suggested in our measured *n* and *μ* values (see Fig. 6(b) and (c), respectively). To clearly present the key properties, Table 1 summarizes the measured *n*, *μ*, *σ*, *S*, *S*^2^*σ*, *C*_p_, and *κ* values of Cu-doped SnSe at both room temperature (300 K) and high temperature (873 K). As can be seen, with increasing the Cu doping level, *n* is drastically enhanced by roughly one order of magnitude from 1.82 × 10^17^ to 3.44 × 10^18^ cm^–3^ at room temperature, resulting in an obvious *σ* enhancement. This is because with an increase in the Cu doping level, the proportion of Cu^+^ in the SnSe system is increased, resulting in the rise in *n*. For *μ*, Fig. 6(c) indicates the relationship with *T*. In fact, the relation of the power law (*μ* ∝ *T*^*d*^) governs the variation of *μ* as a function of *T*.^46^,^52^ There are two regions for *μ*. In the first region, from 300–673 K, *μ* decreases with *T* roughly following the curves related to *μ* ∝ *T*^–1.5^, even though the curves fluctuate more for Cu-doped SnSe, indicating that the scattering mechanism should still be dominated by acoustic phonon scattering.^46^,^52^ In the second region, at high temperatures of 673–873 K, *μ* increases with *T* roughly following the curves related to *μ* ∝ *T*^2.3^, which contributes to higher electrical transport properties above 673 K, indicating that an additional scattering mechanism should exist.^46^,^52^ Previous studies have shown that potential barrier scattering at grain boundaries and/or crystal defects combined with phonon scattering may cause such a special *μ* ∝ *T*^*d*^ relationship.^46^,^53^,^54^ Considering that our Cu-doped SnSe has intensive crystal defects, these results are reasonable. Meanwhile, an increase in the Cu doping level, *μ* decreases gradually, which should be derived from the lattice distortion in SnSe, which scatters the transport of carriers. Fig. 6(d) shows the measured temperature-dependent *S* values for pellets with different *x* values, in which giant *S* values can be observed within the moderate temperature range (from 450 to 700 K), similar to the case for SnSe single crystals.^8^ The peak *S* value found in single crystals (∼600 μV K^–1^ at 525 K along the *a*-axis)^8^ is slightly lower than our peak *S* value (∼700 μV K^–1^ at 523 K with *x* = 0.01). Such peak *S* values come from the bipolar transport.^51^ With increasing *x*, the bipolar transport occurring shifts slightly to a higher temperature, indicating the increase of *n*. Fig. 6(e) shows the determined temperature-dependent *S*^2^*σ* data for pellets with different *x* values. It is clear to see that the *σ* values play a dominant role in determining *S*^2^*σ*, and the peak *S*^2^*σ* value of 5.57 μW cm^–1^ K^–2^ can be found at high temperature (823 K) in the Sn_0.882_Cu_0.118_Se pellet.

**Table 1:** The *ρ*, *n*, *μ*, *σ*, *S*, *S*^2^*σ*, *C*_p_, and *κ* of Cu-doped SnSe for *x* = 0, 0.01, 0.02, 0.05, 0.075, 0.1 and 0.118 at both room temperature (300 K) and high temperature (873 K)

Parameter	*x* = 0	*x* = 0.01	*x* = 0.02	*x* = 0.05	*x* = 0.075	*x* = 0.1	*x* = 0.118
*ρ* (g cm^–3^)	6.084	6.068	6.089	6.112	6.125	6.14	6.142
*n* (cm^–3^) at 300 K	1.82 × 10^17^	2.84 × 10^17^	4.63 × 10^17^	9.48 × 10^17^	1.7 × 10^18^	2.94 × 10^18^	3.44 × 10^18^
*n* (cm^–3^) at 873 K	1.81 × 10^18^	1.60 × 10^18^	2.31 × 10^18^	3.86 × 10^18^	6.48 × 10^18^	1.58 × 10^19^	2.04 × 10^19^
*μ* (cm^2^ V^–1^ s^–1^) at 300 K	160.7	112.9	85.6	76.2	71.6	58.3	57.2
*μ* (cm^2^ V^–1^ s^–1^) at 873 K	34.0	30.2	28.5	27.7	24.5	18.7	16.4
*σ* (S cm^–1^) at 300 K	4.7	5.1	6.4	11.6	19.5	27.4	31.6
*σ* (S cm^–1^) at 873 K	9.9	7.7	10.5	17.1	25.4	47.5	53.7
*S* (μV K^–1^) at 300 K	504.6	481.8	458.4	408.1	355.3	297.9	282.7
*S* (μV K^–1^) at 873 K	433.5	422.3	406.2	387.2	356.8	324.6	315.3
*S* ^2^ *σ* (μW cm^–1^ K^–2^) at 300 K	1.19	1.2	1.33	1.93	2.46	2.43	2.52
*S* ^2^ *σ* (μW cm^–1^ K^–2^) at 873 K	1.85	1.38	1.74	2.57	3.24	5.01	5.34
*C*_p_ (J g^–1^ K^–1^) at 300 K	0.273	0.272	0.270	0.260	0.257	0.253	0.252
*C*_p_ (J g^–1^ K^–1^) at 873 K	0.288	0.286	0.283	0.272	0.267	0.264	0.262
*κ* (W m^–1^ K^–1^) at 300 K	0.93	0.86	0.82	0.73	0.68	0.62	0.58
*κ* (W m^–1^ K^–1^) at 873 K	0.47	0.45	0.43	0.4	0.36	0.33	0.33

**Fig. 7:**
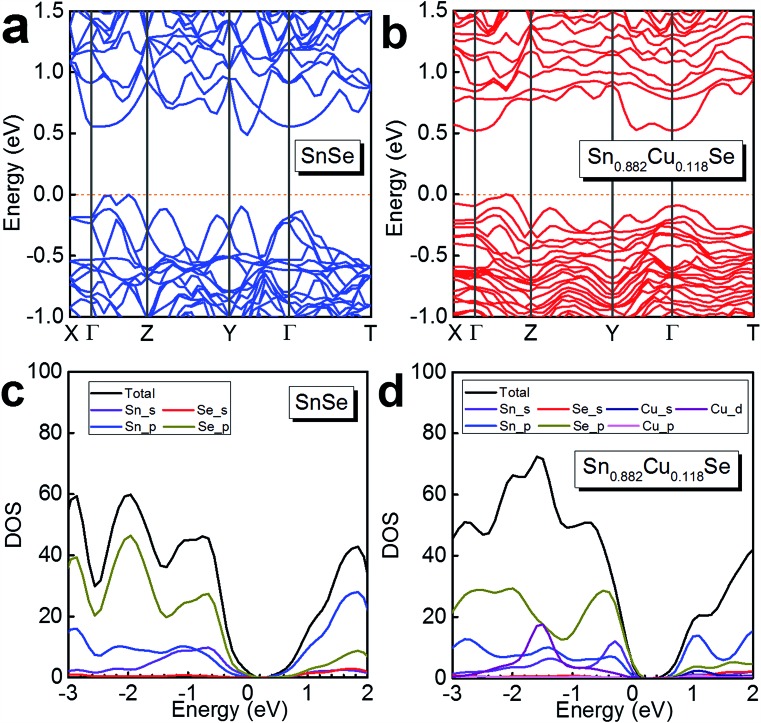
Calculated band structure of (a) SnSe and (b) Sn_0.882_Cu_0.118_Se, and the calculated density of states (DOS) of (c) SnSe and (d) Sn_0.882_Cu_0.118_Se.

To further understand the electrical transport properties of our heavily Cu-doped SnSe, we performed density function theory (DFT) calculations to illustrate the evolution of the band structure of SnSe after Cu-doping. Fig. 7(a) and (b) show the calculated band structures of SnSe before and after heavy Cu-doping, respectively, and the valence band maxima are both pinned to 0 eV in energy. For pure SnSe, as shown in Fig. 7(a), two distinct conduction band minima can be observed around *Y* and *Γ* points of the Brillouin zone, which are denoted as CB_1_ and CB_2_, respectively. For the valence band, six maxima can be clearly depicted, with two principal ones lying along the *Γ*–*Z* line. For the heavily Cu-doped SnSe, as shown in Fig. 7(b), there are also two distinct conduction band minima around the *Y* and *Γ* points of the Brillouin zone, denoted as CB_1_ and CB_2_. However, for the valence band, different from the pure SnSe, the maxima is not as sharp as pure SnSe, and obvious band convergence of multiple-valences can be observed after heavy Cu-doping, which is responsible for the enhanced *S*^2^*σ*.^2^,^55^ Fig. 7(c) and (d) show the calculated density of states (DOS) of SnSe before and after heavy Cu-doping, respectively. Taking Fig. 7(a) and (b) into the consideration, it is clear that the doped Cu (mainly by Cu_d) enhances the DOS at the valence bands, indicating the increase of *n*, agreeing with the experimental results. Overall, the heavy Cu-doping can significantly improve the hole concentration in SnSe and result in an enhanced *S*^2^*σ*.

By using *κ* = *DC*_p_*ρ*,^10^ the temperature-dependent *κ* values for pellets with different *x* values can be calculated and plotted, as in Fig. 6(f). The *D* values are plotted in the inset of Fig. 6(f) as a reference, and the measured *C*_p_ and *ρ* values are also listed in Table 1. With increasing *x*, *κ* decreases gradually, which could be derived from increased lattice distortions in SnSe, which contribute to effective phonon scatterings. A low *κ* of 0.32 W m^–1^ K^–1^ is achieved at 823 K in the Sn_0.882_Cu_0.118_Se pellet. Because the densities of our sintered pellets are relatively high (all >98.2%),^8^,^14^ these *κ* values are close to the intrinsic value of isotropic SnSe. To understand the observed low *κ* in our pellets, we investigated the lattice contributions (*κ*_l_) and electrical contributions (*κ*_e_). *κ*_e_ and *κ*_l_ are determined by *κ*_e_ = *LσT* and *κ*_l_ = *κ* – *κ*_e_ according to the Wiedemann–Franz law,^56^ where *L* is the Lorenz number and *L* ≈ 1.5 × 10^–8^ V^2^ K^–2^ is used in this study, as calculated using the single parabolic band model^57^–^59^ as shown with calculation details in Section 6 of the ESI.† In fact, *L* = 1.5 × 10^–8^ V^2^ K^–2^ has been widely used previously since, for SnSe, the *κ* significantly depends on phonon scattering.^2^,^8^,^10^,^16^ Fig. 6(g) shows plots of the determined temperature-dependent *κ*_e_ for pellets with different *x* values, in which the obtained *σ* shown in Fig. 6(a) were used for determining *κ*_e_. Our obtained *κ*_e_ values possess the same trend as for *σ*, but the values are very low (all <0.05 W m^–1^ K^–1^ over the entire temperature range). Fig. 6(h) plots *κ*_l_ using *κ*_l_ = *κ* – *κ*_e_ for pellets with different *x* values, where all of the *κ*_l_ values are significantly low, in particular, only ∼0.25 W m^–1^ K^–1^ at 823 K for *x* = 0.118. It should also be noticed that our achieved *κ*_l_ value is close to the calculated minimum *κ*_l_ (*κ*_l min_) *via* a classical Debye–Cahill model,^60^ from which the calculated *κ*_l min_ were 0.26, 0.36 and 0.33 W m^–1^ K^–1^ along the *a*-, *b*- and *c*-axis,^8^,^17^ respectively. In fact, because this calculation is based on the intrinsic SnSe without doping and an ideal relative density of 100%, our achieved *κ*_l_ values are slightly lower than the calculated *κ*_l min_, which is reasonable. The inset of Fig. 6(h) shows the plots of *κ*_l_ as a function of 1000/*T* for pellets with different *x* values and all show a linear relationship, indicating that the phonon scatterings are dominated by the Umklapp phonon scattering.^61^,^62^ Such low *κ*_l_ values are attributed to the strongly anharmonic bonding,^8^,^62^–^67^ as well as crystal imperfections such as the lattice distortions caused by local non-uniform doping and dislocations and grain boundaries (or interfaces).^68^,^69^ The calculated *κ*_l_/*κ* ratio for our pellets are all greater than 80%, indicating that the phonon transport dominates the *κ* values, as shown in Fig. S6(b) in the ESI.† Fig. 6(i) shows a comparison of experimental *ZT* values with predicted values by calculation at 823 K, where the calculation was based on a single parabolic band model (detailed calculations can be seen in Section 6 of the ESI†).^57^–^59^,^70^ It is clear to see that our measured *n* value (2.04 × 10^19^ cm^–3^) is very close to the predicted value (∼3 × 10^19^ cm^–3^), which can result in a peak *ZT* of ∼1.5, indicating that there is still scope for achieving a higher *ZT*.

**Fig. 8:**
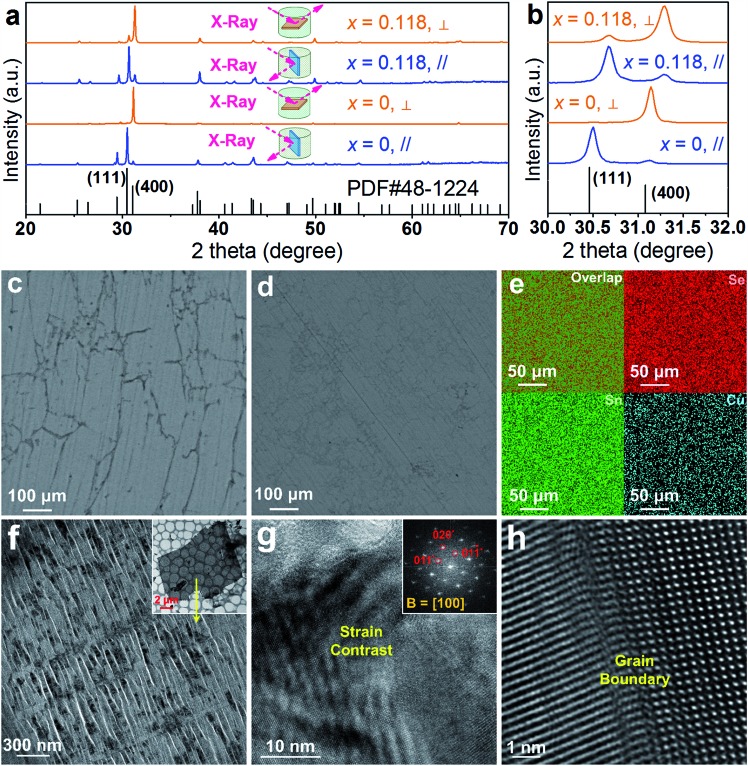
(a) XRD patterns of sintered SnSe pellets (both pure SnSe and Sn_0.882_Cu_0.118_Se) measured along both the ⊥ (yellow line) and ∥ (blue line) direction. (b) Magnified XRD patterns to see the peak deviation at 111* and 400*. SEM images of polished surfaces taken from sintered (c) SnSe and (d) Sn_0.882_Cu_0.118_Se pellets. (e) Corresponding EDS mapping results for (d). (f) Magnified TEM image taken from a laminar TEM specimen sliced using an ultramicrotome as shown in the inserted TEM image. (g) [100] zone-axis HRTEM image with inserted FFT pattern to show strain contrast. (h) HRTEM image to show a grain boundary.

To further understand the low *κ*_l_ data, we analysed our sintered pellets by XRD, SEM and TEM characterizations, and the results are shown in Fig. 8. Fig. 8(a) shows typical XRD results for both pure SnSe and Sn_0.882_Cu_0.118_Se pellets; here, all diffraction peaks for all sintered pellets can be exclusively indexed as the orthorhombic structured SnSe, and a space group of *Pnma* (Standard Identification Card, JCPDS 48-1224), indicating that the compositional features were successfully retained after sintering and no other phase was observed. Fig. 8(b) shows the magnified XRD patterns and demonstrates the peak deviation at 111* and 400*, from which the samples cut along the ⊥ direction show a strong 400* peak, and the samples cut along the ∥ direction shows a strong 111* peak. Comparing the XRD results of the two pellets, it is clear that the 111* peak of Sn_0.882_Cu_0.118_Se is much stronger than that of pure SnSe along the ⊥ direction, and the 400* peak of Sn_0.882_Cu_0.118_Se is also stronger than that of pure SnSe along the ∥ direction, both indicating that Sn_0.882_Cu_0.118_Se pellets possess a much weaker anisotropy than pure SnSe pellets. Besides, compared with the pure SnSe pellets, the 111* and 400* peaks from the Sn_0.882_Cu_0.118_Se pellets shift towards a higher 2*θ*, indicating that Cu atoms are still incorporated into the SnSe lattice. Fig. 8(c) and (d) show SEM images of polished surfaces taken from sintered pure SnSe and Sn_0.882_Cu_0.118_Se pellets along their ⊥ directions, respectively. The comparison indicates that the Cu-doped SnSe pellet possesses a much smaller grain size than that of the pure SnSe pellet, derived from the difference of their product sizes before sintering. Besides, this comparison also explains why the anisotropy of the thermoelectric performance for the Sn_0.882_Cu_0.118_Se pellet becomes weaker than that of the pure one. Fig. 8(e) shows the corresponding EDS map results for the Sn_0.882_Cu_0.118_Se pellet, in which all elements are uniform at a microscale, indicating the stability of the compositions before and after sintering. Through detailed EPMA studies on the Sn_0.882_Cu_0.118_Se pellet, the ratio of Sn : Cu : Se was measured as 44.12 : 5.89 : 49.99, indicating the stable composition of Sn_0.882_Cu_0.118_Se. Fig. 8(f) is a magnified TEM image taken from a laminar TEM specimen sliced using an ultramicrotome (inset TEM image in Fig. 8(f)), in which cracks (fractured during ultramicrotome processing) can be seen due to the weak van der Waals force between the Sn–Se layers. Nevertheless, crystals can be seen between the cracks, which can be used to evaluate the structural characteristics of the sintered pellets. Fig. 8(g) is a [100] zone-axis HRTEM image with inset the fast Fourier transform (FFT) pattern, where strain contrast is observed. Fig. 8(h) is another HRTEM image taken from a typical grain boundary. Such local structural variations cause lattice distortions, which in turn enhance the phonon scatterings and contribute to the low *κ*_l_ values. All these results demonstrate that the compositional and structural features have been successfully retained during the sintering. In fact, the “intensive crystal imperfections” were derived from the synthesis, which were shown in Fig. 3 and 4.

To compare the thermoelectric properties in more detail, Table 2 summarizes the main thermoelectric properties, including *ZT*, *σ*, *S*, *S*^2^*σ*, *κ*, *n* and *ρ* with the similar studies of p-type doped SnSe. As can been seen, the low *κ* and moderate *σ* values play the dominant role in achieving the competitive high *ZT* figures in our heavily Cu-doped SnSe. Section 7 in the ESI† also summarizes both average and peak *ZT* values with the similar studies of p-type doped SnSe, indicating that our heavily Cu-doped SnSe is very competitive.

**Table 2:** Comprehensive summary of the thermoelectric performance of p-type doped polycrystalline SnSe. Here, solvothermal is abbreviated as ST, hydrothermal is abbreviated as HT, melting is abbreviated as M, zone-melting is abbreviated as ZM, annealing is abbreviated as A, solid-state solution is abbreviated as SSR, mechanical alloying is abbreviated as MA, hot-pressing is abbreviated as HP, and spark plasma sintering is abbreviated as SPS. The * means that the *n* values were measured at room temperature

Product	Synthetic method	*ZT*	*T* (K)	*σ* (S cm^–1^)	*S* (μV K^–1^)	*S* ^2^ *σ* (mW m^–1^ K^–2^)	*κ* (W m^–1^ K^–1^)	*n* (10^19^ cm^–3^)	*ρ* (g cm^–3^)	Ref.
11.8% Cu-doped SnSe	ST + SPS	1.41	823	∼55.9	∼315.6	∼0.57	∼0.32	1.95	∼6.14	This work
Sn_0.97_Cu_0.03_Se	M + HP	0.79	823	∼35.0	∼325.1	∼0.37	∼0.39	∼0.016*	6.16	31
Sn_0.98_Cu_0.02_Se	M + A + SPS	0.7	773	∼42.4	∼238.6	∼0.24	0.27	18.4*	∼6.12	29
Sn_0.99_Cu_0.01_Se	HT + HP	1.2	873	∼36.4	∼313.8	∼0.35	∼0.2	—	—	71
Sn_0.99_Ag_0.01_Se	M + A + HP	0.6	750	∼45.9	∼344.1	∼0.54	∼0.68	∼0.35*	∼5.93	33
Sn_0.99_Ag_0.01_Se	M + A + SPS	0.74	823	∼54.8	∼330.9	0.6	∼0.66	1.9*	∼5.99	32
Sn_0.985_Ag_0.015_Se	M	1.3	773	∼44.7	∼344.0	∼0.52	∼0.30	∼0.8*	5.87	35
Sn_0.97_Ag_0.03_Se	ST + SPS	0.8	850	∼90.3	∼266.2	∼0.64	∼0.68	0.9*	>5.56	36
Sn_0.99_Na_0.01_Se	M + A + SPS	0.85	800	∼100.4	∼271.5	∼0.74	∼0.50	∼6.5*	5.94	24
Sn_0.99_Na_0.01_Se	M + SPS	0.75	823	∼49.6	∼311.1	0.48	∼0.53	1.0*	∼5.99	22
Sn_0.99_Na_0.01_Se	M + SPS	∼0.8	800	∼81.2	∼267.2	∼0.58	∼0.50	∼1.5	—	21
Sn_0.985_Na_0.015_Se	M + MA + HP	∼0.8	773	∼37.9	∼298.8	∼0.34	∼0.33	∼2.1*	5.81	23
Sn_0.98_Na_0.02_Se	SPS	0.87	798	∼56.4	∼288.8	0.47	0.4	3.08*	∼5.81	26
Sn_0.97_Na_0.03_Se	SPS	0.82	773	∼65.1	∼280.2	∼0.51	∼0.50	∼2.2	∼5.93	72
Sn_0.99_Na_0.005_K_0.005_Se	MA + SPS	1.2	773	∼34.9	∼374.7	∼0.49	0.32	∼7.2*	5.71	25
Sn_0.995_Na_0.005_SeCl_0.005_	SSR + HP	0.84	810	∼79.2	∼228.6	∼0.41	∼0.39	∼3.95*	∼5.93	73
Sn_0.99_Na_0.01_Se_0.84_Te_0.16_	MA + SPS	0.72	773	∼67.4	∼275.0	∼0.51	∼0.50	—	—	74
(Sn_0.96_Pb_0.04_)_0.99_Na_0.01_Se	M + SPS	∼1.2	773	∼89.4	∼269.7	∼0.65	∼0.45	∼2.8	—	27
Sn_0.99_K_0.01_Se	MA + SPS	∼1.1	773	∼18.6	∼421.4	∼0.33	∼0.24	0.92*	—	28
Sn_0.995_Tl_0.005_Se	M + HP	0.6	725	∼68.9	∼300.0	∼0.62	∼0.75	—	∼5.99	75
Sn_0.99_In_0.01_Se	M + HP	0.2	823	∼6.53	∼350.0	∼0.08	∼0.36	∼0.03*	∼5.87	76
Sn_0.9_Ge_0.1_Se	M	—	400	—	∼843.2	—	∼0.39	—	—	77
Sn_0.96_Ge_0.04_Se	ZM + HP	0.6	823	35.6	∼378.5	0.51	∼0.7	∼0.03*	>5.81	78
Sn_0.99_Zn_0.01_Se	M + HP	0.96	873	∼74.1	∼328.5	0.8	∼0.73	∼0.45	—	79
Sn_0.97_Sm_0.03_Se	M + HP	0.55	823	∼33.6	∼250.0	∼0.21	∼0.32	∼0.013*	—	42
SnSe_0.985_Cl_0.015_	M	1.1	773	∼25.5	∼399.3	∼0.41	∼0.30	∼0.01*	5.87	35
SnSe_0.9_Te_0.1_	ST + SPS	1.1	800	∼57.4	∼322.8	∼0.60	∼0.44	∼1*	∼5.87	55

## Conclusions

In conclusion, a high doping limit of Cu at 11.8% has been achieved in single-crystal Cu-doped SnSe microbelts for the first time synthesized *via* a facile solvothermal method. Through detailed structural and chemical characterizations, with increasing the Cu doping level, the morphology of Cu-doped SnSe transfers from rectangular plates to microbelts. Both Cu^+^ and Cu^2+^ co-exist in the microbelts. Lattice distortions are observed, which play a dominant role in keeping the heavily doped SnSe microbelts as an orthorhombic structure. Besides, the pellets sintered from such heavily Cu-doped microbelts demonstrate a high thermoelectric performance. The high *ZT* value of ∼1.41 at 823 K was achieved, coming from the high power factor and low thermal conductivity. This study fills in the gaps of the existing knowledge concerning the doping mechanisms of Cu in SnSe systems, and provides a new strategy for achieving a high thermoelectric performance in SnSe-based thermoelectric materials.

## Experimental section

### General procedures and materials

The precursors include SnCl_2_·2H_2_O (99.99%), Na_2_SeO_3_ (99.99%), CuCl_2_ (99.99%), ethylene glycol anhydrous (99.8%), and NaOH (99.99%), all of which were purchased from Sigma-Aldrich Co. LLC. The solvothermal reactions can be expressed as:^46^1
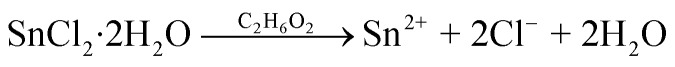

2
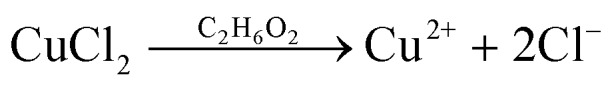

3
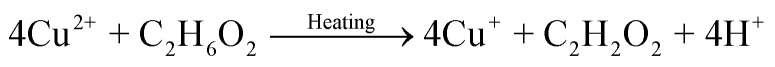

4
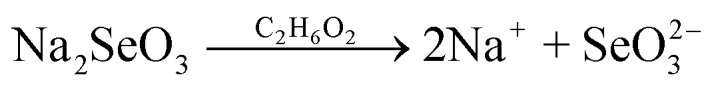

5 SeO_3_^2–^ + C_2_H_6_O_2_ → Se + C_2_H_2_O_2_ + H_2_O + 2OH^–^

6 Se + Sn^2+^ → Se^2–^ + Sn^4+^

7 (1–*x*–*y*)Sn^2+^ + *x*Cu^2+^ + *y*Cu^+^ + Se^2–^ → Sn_1–*x*–*y*_Cu_*x*+*y*_Se (not balanced).

Here NaOH (99.99%) was used to adjust the environment of the solvent, and ethylene glycol (EG, 45 ml) acted as both the solvent and the reducing agent, which benefited the ion reaction.^46^,^80^,^81^ The solution was kept stirring for 10 min at room temperature, before being sealed in a polytetrafluoroethylene-lined stainless steel autoclave (125 ml). The autoclave was heated at 230 °C for 36 h in an oven, followed by furnace cooling to room temperature. The synthesized products were collected by centrifugation, and the secondary phase (Cu_2_Se) was removed *via* ultrasonic-assisted sedimentation. The purified products were then washed using ethanol and deionized water several times, before drying in the oven at 60 °C for 15 h.

### Instruments

The synthesized products were characterized by XRD (Bruker-D8) to determine their crystal structures, and by XPS (Kratos Axis Ultra) to determine the valence state of Cu in the SnSe (the energy scale was calibrated by carbon). Lattice parameters were obtained by analysing the diffraction patterns using the JADE software package. EPMA (JEOL JXA-8200) was used to determine their compositions. SEM (JSM-6610, JEOL Ltd.) was used to obtain the morphological characteristics of the synthesized products, and HR-TEM (TECNAI-F20) and Cs-corrected HR-STEM (Titan-G2) were used to characterize their structural and chemical features. The TEM specimens of the sintered samples were prepared by slicing the sample using an ultramicrotome.

### Property measurement

The synthesized products were sintered by spark plasma sintering (SPS, SPS-211Lx, Fuji Electronic Co., Ltd.) with a pressure of 60 MPa at 900 K for 5 min to form disc-shaped pellets with dimensions of *∅* = 12.6 mm and *h* = 8.0 mm. The Archimedes method was used to measure the density *ρ*. A Seebeck coefficient/electric resistivity measuring system (ZEM-3, ULVAC Technologies, Inc.) was used to simultaneously measure *σ* and *S* between 300 and 873 K. The laser flash diffusivity method (LFA 457, NETZSCH Group) was used to measure the thermal diffusivity *D*, and *κ* was calculated by *κ* = *DC*_p_*ρ*,^8^ where *C*_p_ is the specific heat capacity obtained by differential scanning calorimetry (DSC 404 C; NETZSCH Group). The van der Pauw technique was used to measure *n* under a reversible magnetic field of 1.5 T. Each pellet is measured at least three times to ensure the repeatability of their thermoelectric properties. The measured repeatability is achieved with fluctuations of *σ*, *S* and *κ* being 10%, 1.5% and 5%, respectively, as shown in Fig. S8 of the ESI.†

### Density functional theory (DFT) calculations

DFT calculations were based on the full potential linearized augmented plane-wave (FP-LAPW) method^55^ implemented in the WIEN2K code.^55^ Supercells of 2 × 2 × 2 unit cells of SnSe were built for the purpose of randomly replacing Se sites with Cu atoms. The generalized gradient approximation (GGA) and the Perdew–Burke–Ernzerhof (PBE) functional were used to describe the exchange and correlation interactions.^55^ The electronic band structures and the density of states values were calculated after self-consistency cycle calculation with the convergence criteria set as energy less than 0.0001 Ry and the leaking charge less than 0.0001 eV.

## Conflicts of interest

There are no conflicts to declare.

## Supplementary Material

Supplementary information
