# Supplementary material for: Boosting the thermoelectric performance of p-type heavily Cu-doped polycrystalline SnSe via inducing intensive crystal imperfections and defect phonon scattering
Source: Chem Sci. 2018 Jul 30;9(37):7376–89. doi: 10.1039/c8sc02397b (PMC6237129; doi:10.1039/c8sc02397b)
Supplement: Supplementary file 1 [file SC-009-C8SC02397B-s001.pdf]

Electronic Supplementary Material (ESI) for Chemical Science.
This journal is © The Royal Society of Chemistry 2018

Supporting Information

Section 1. Demonstration of removing Cu_2Se secondary phase.

To make a clear claim for the demonstration of removing Cu_2Se secondary phase, we add three detailed XRD patterns of synthesized products with r = 10 %, r = 20 % with Cu_2Se secondary phase, and r = 20 % after removing the Cu_2Se secondary phase, respectively, as shown in Figure S1(a-c). It can be seen that when r = 10 %, there is no Cu_2Se secondary phase, indicating that it has not reached the Cu solubility. Figure S1(b) shows that Cu_2Se secondary phase has been observed when r = 20 % and indicate the products have reached the Cu solubility. The Cu solubility was analyzed to be 11.8 % via electron probe micro-analysis (EPMA). Also, when r = 20 % after removing the Cu_2Se secondary phase, no Cu_2Se can be found, indicating that high pure SnSe phase can be achieved by our solvothermal method.

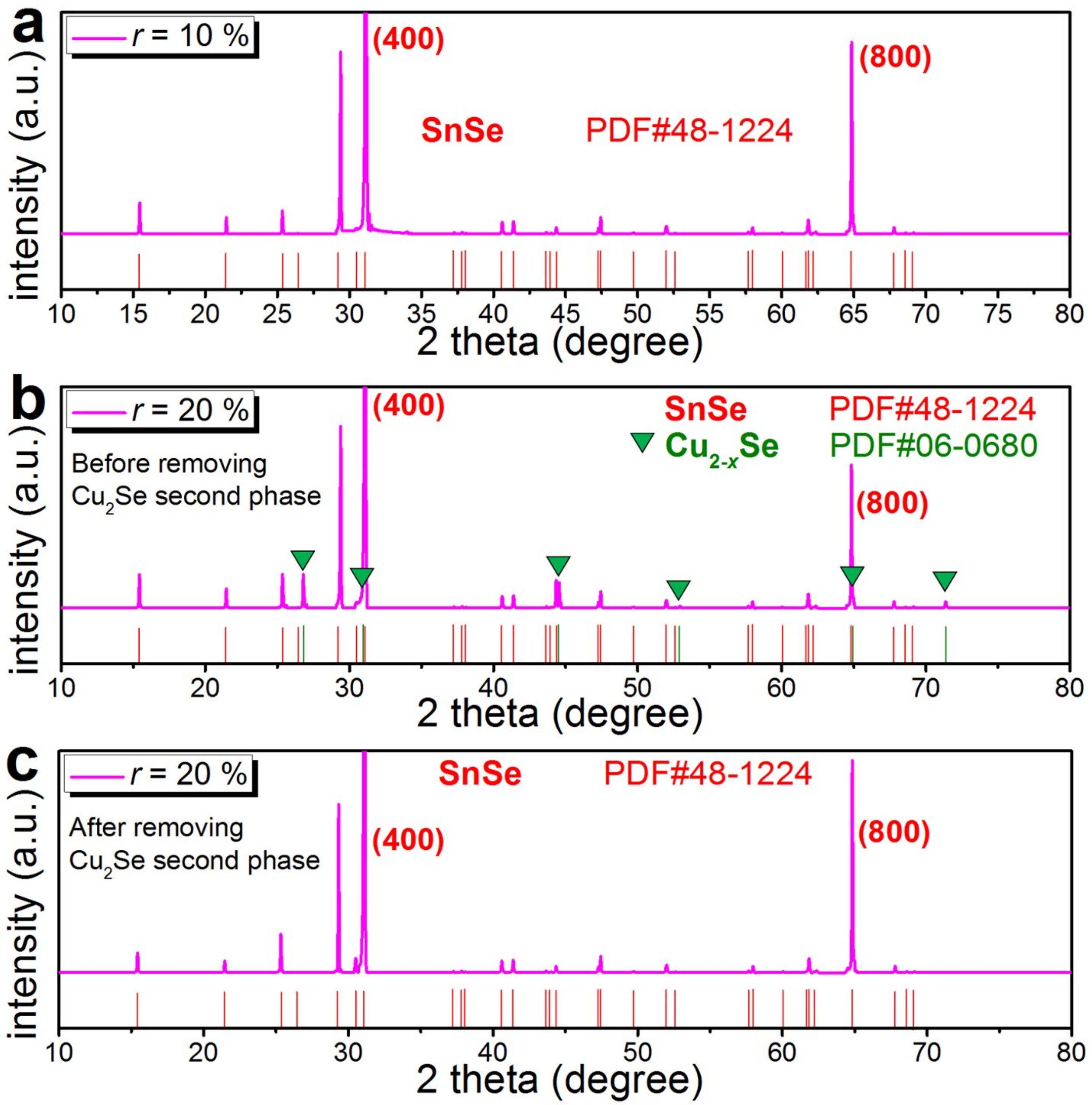

Figure S1. Detailed XRD patterns of synthesized products with (a) r = 10 %, (b) r = 20 % before removing Cu_2Se secondary phase, (c) r = 20 % after removing Cu_2Se secondary phase.

Section 2. Transitions of lattice constant and unit cell volume.

Figure S2(a-c) are the plots of the calculated lattice parameters (*a*, *b*, and *c*). As can be seen, with increasing *r*, both *a* and *b* decrease until the solubility (11.8 %) of Cu in SnSe is reached. For *c*, however, the value slightly fluctuates in the entire range of *r*. This result suggests that there may be special atomic arrangement in our heavily Cu-doped SnSe, resulting in a morphology change for the single crystal products. From determined lattice parameters shown Figure S2(a-c), the unit cell volumes for different *r* can be calculated. Figure S2(d) shows the result, in which the unit cell shrinks is found with increasing *r*. Considered that the radius of Cu^{+} (0.077 nm) or Cu^{2+} (0.073 nm) are much smaller than that of Sn^{2+} (0.112 nm) and Se^{2-} (0.198 nm), the unit cell shrinkage is expected.

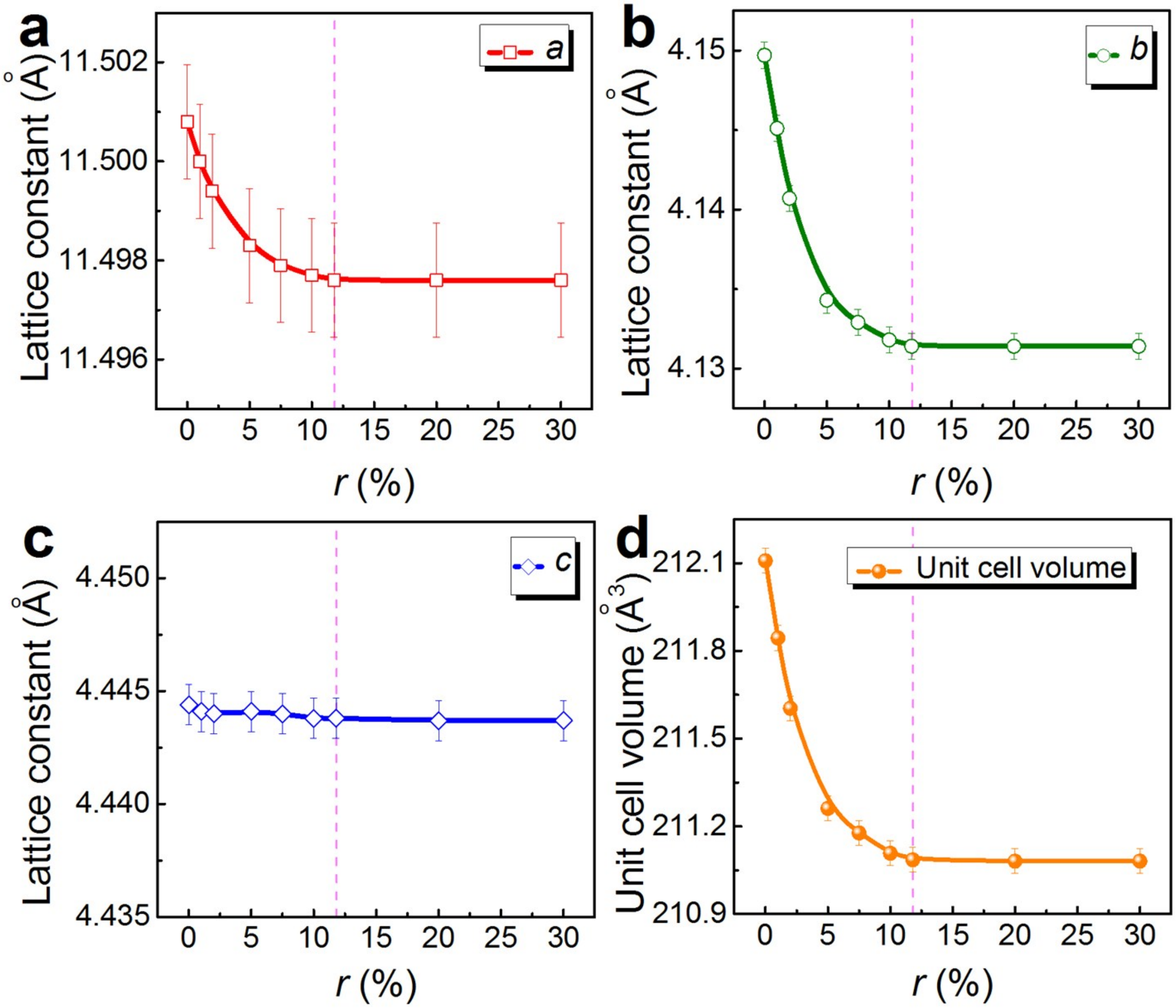

Figure S2. The determined *r*-dependent lattice parameters (a) *a*, (b) *b*, (c) *c*, and (d) unit cell volumes. The dashed line indicates the solubility of Cu.

Section 3. Detailed morphological transition.

To demonstrate the morphological transition of synthesized products with increasing x values, intensive SEM investigations are performed. Figure S3(a-f) show the SEM images of synthesized products for $x = 0$, $x = 0.01$, $x = 0.05$, $x = 0.1$, $x = 0.118$ when $r = 20$ %, and $x = 0.118$ when $r = 30$ %, respectively. It is clear to see that with increasing x, the morphology of SnSe products gradually transfers from rectangular plate-like into long belt-like morphology. Meanwhile, when x reaches to 0.118, the morphology does not change, indicating the solubility of Cu.

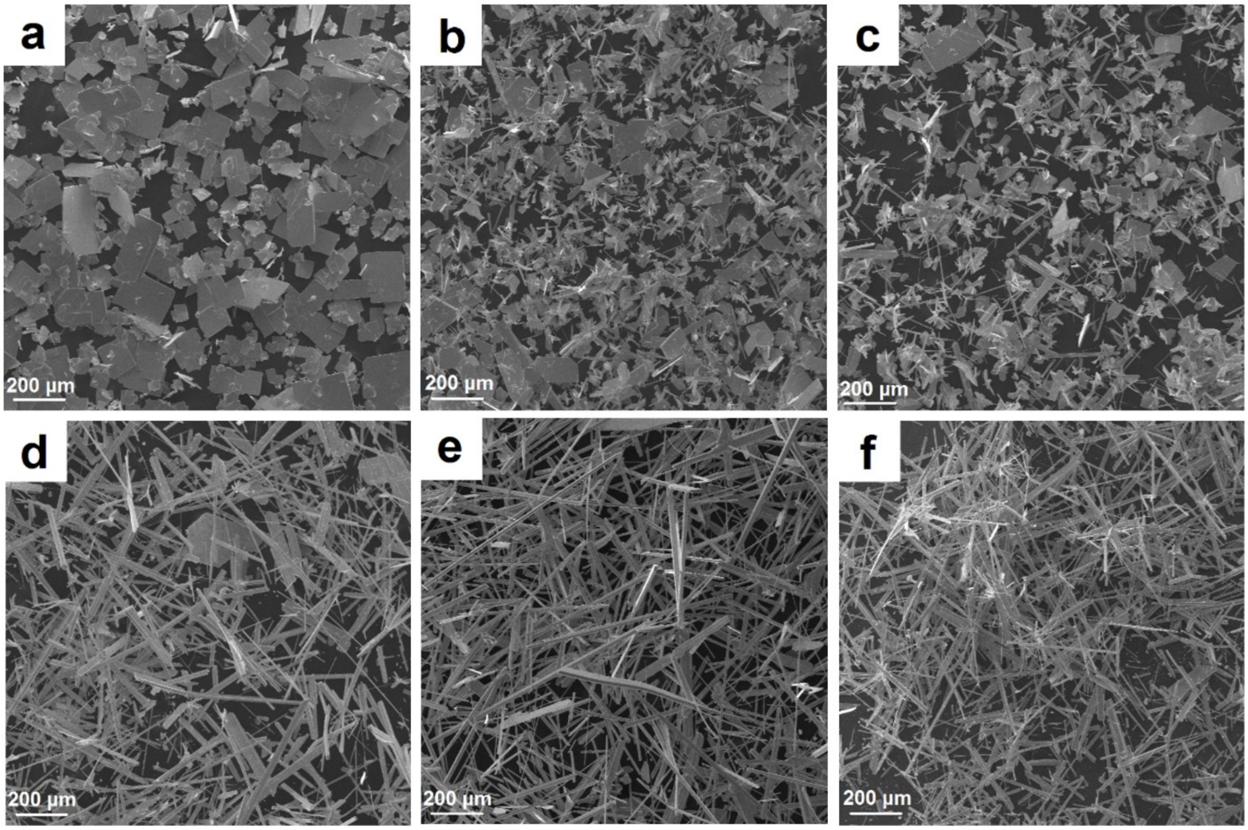

Figure S3. SEM images of synthesized products for (a) $x = 0$, (b) $x = 0.01$, (c) $x = 0.05$, (d) $x = 0.1$, (e) $x = 0.118$ when $r = 20$ %, and (f) $x = 0.118$ when $r = 30$ %, respectively.

Section 4. Simulation of crystals.

To illustrate the potential surfaces of our SnSe plate and Cu-doped SnSe belt, we simulate the single crystal plate of SnSe via using software (WinXMorph) [1], and corresponding crystal model is shown in Figure S4(a-b). It needs to be emphasized that the real heavily Cu-doped SnSe is a microbelt, not a plate like (b) shows.

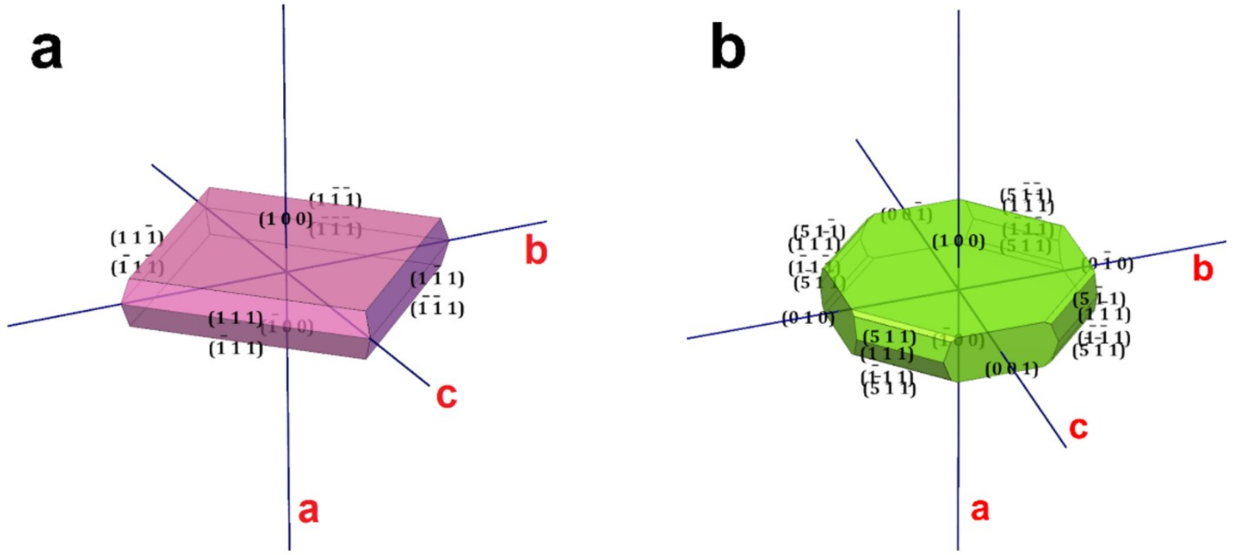

Figure S4. Crystal model of (a) undoped and (b) heavily Cu-doped SnSe with labelled crystallographic plane.

Section 5. Anisotropy of thermoelectric performance.

To demonstrate the anisotropy of thermoelectric performance in our SnSe pellets, we show the plots of properties (temperature-dependent *σ*, *S*, $S^2\sigma$, *κ*, and *ZT*) with different measured directions from the SnSe and $Sn_{0.882}Cu_{0.118}Se$ pellets in Figure S5, in which label "⊥" stands for the measured directions perpendicular to the sintering pressure, and label "//" represents the measured directions parallel to the sintering pressure. It is clear that except *S*, all properties measured along the ⊥ direction are higher than that measured along the // direction due to the anisotropy. This is why we chose ⊥ direction as the main measured direction.

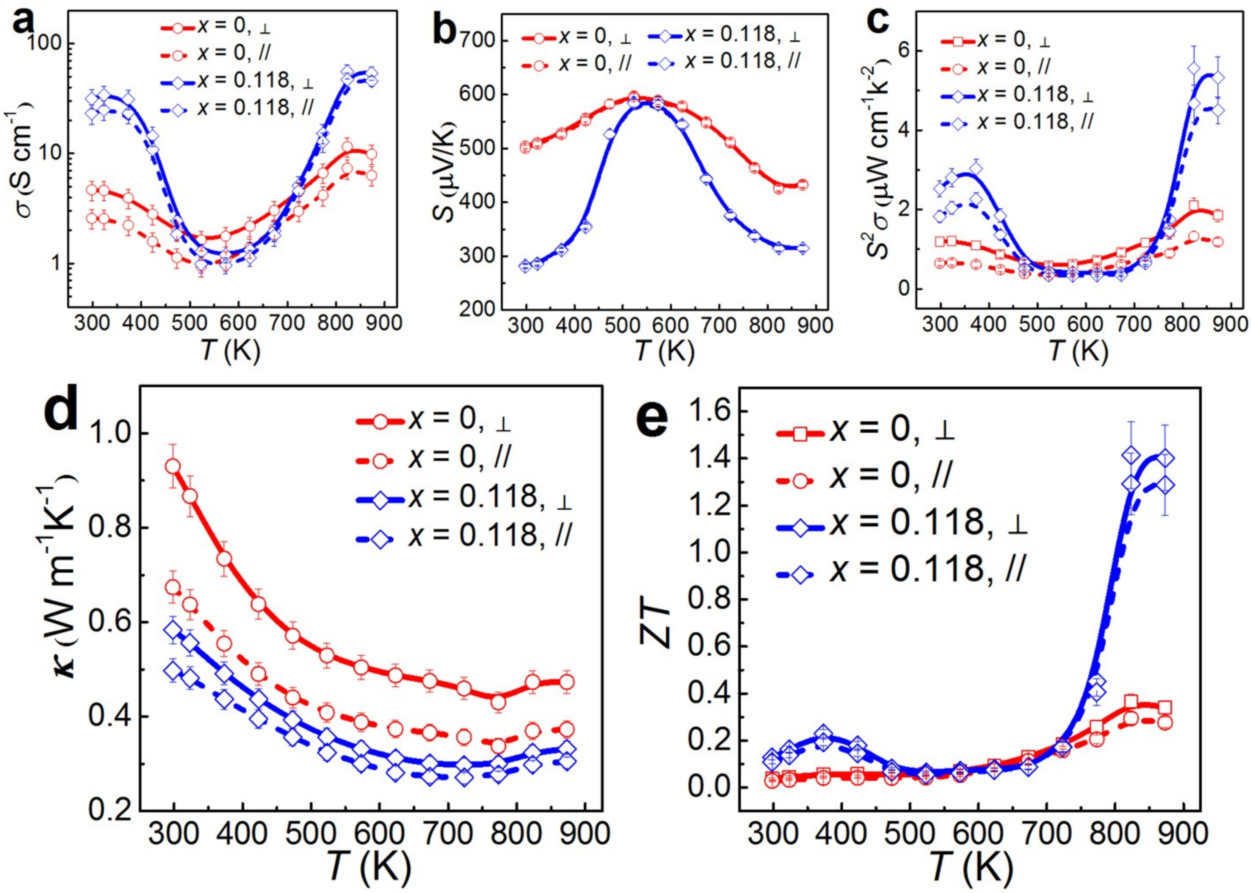

Figure S5. Plots of *T*-dependent properties with different measured directions from our SnSe pellets with different *x* values: (a) *σ*; (b) *S*; (c) $S^2\sigma$; (d) *κ*, and (e) *ZT*. The measured directions perpendicular to the sintering pressure is labelled as "⊥", and the measured directions parallel to the sintering pressure is labelled as "//".

Section 6. Calculated Lorenz number and ratio of *T*-dependent κ_l/κ for Cu-doped SnSe pellets.

The calculated Lorenz number (*L*) for Cu-doped SnSe pellets are shown in Figure S6(a). The *L* values for all sintered pellets are stable with the entire temperature range (~1.5×10^{-8} V^2 K^{-2}), indicating that SnSe is a typical thermoelectric material that the κ are significantly depended on the phonon scattering. For calculation details, a single parabolic band model[2-4] was employed as:

$$S(\eta) = \frac{k_B}{e} \cdot \left[\frac{\left(r + \frac{5}{2}\right) \cdot F_{r+\frac{3}{2}}(\eta)}{\left(r + \frac{3}{2}\right) \cdot F_{r+\frac{1}{2}}(\eta)} - \eta \right] \quad \text{(S6-1)}$$

$$n = \frac{1}{e \cdot R_H} = \frac{(2m^* \cdot k_B T)^{\frac{3}{2}}}{3\pi^2 \hbar^3} \cdot \frac{\left(r + \frac{3}{2}\right)^2 \cdot F^2_{r+\frac{1}{2}}(\eta)}{(2r + \frac{3}{2}) \cdot F_{2r+\frac{1}{2}}(\eta)} \quad \text{(S6-2)}$$

$$\mu = \left[\frac{e\pi\hbar^4 \qquad C_l}{\sqrt{2}(k_B T)^{\frac{3}{2}} E_{def}{}^2 (m^*)^{\frac{5}{2}}} \right] \frac{(2r + \frac{3}{2}) \cdot F_{2r+\frac{1}{2}}(\eta)}{\left(r + \frac{3}{2}\right)^2 \cdot F_{r+\frac{1}{2}}(\eta)} \quad \text{(S6-3)}$$

$$L = (\frac{k_B}{e})^2 \cdot \left\{ \frac{\left(r + \frac{7}{2}\right) \cdot F_{r+\frac{5}{2}}(\eta)}{\left(r + \frac{3}{2}\right) \cdot F_{r+\frac{1}{2}}(\eta)} - \left[\frac{\left(r + \frac{5}{2}\right) \cdot F_{r+\frac{3}{2}}(\eta)}{\left(r + \frac{3}{2}\right) \cdot F_{r+\frac{1}{2}}(\eta)} \right]^2 \right\} \quad \text{(S6-4)}$$

where η, k_B, e, r, R_H, $\hbar$, C_l and E_{def} are the reduced Fermi level, the Boltzmann constant, the electron charge, the carrier scattering factor (r = -1/2 for acoustic phonon scattering),[3] the Hall coefficient, the reduced plank constant, the elastic constant for longitudinal vibrations and the deformation potential coefficient, respectively. Here:

$$C_l = v_l^2 \cdot \rho \quad \text{(S6-5)}$$

where v_l is the longitudinal sound velocity and taken as 3350 m·s^{-1} in this study.[5] $F_i(\eta)$ is the Fermi integral expressed as:

$$F_i(\eta) = \int_0^{\infty} \frac{x^i}{1 + e^{(x-\eta)}} dx \quad \text{(S6-6)}$$

The predicted *ZT* values in Figure 6(i) are also calculated based on the above model at 823 K, and the κ_l used in the calculation are derived from Figure 6(h). Figure S6(b) shows the calculated κ_l/κ ratio, in which all ratios are greater than 80 % for our pellets, indicating that the phonon transport dominates κ.

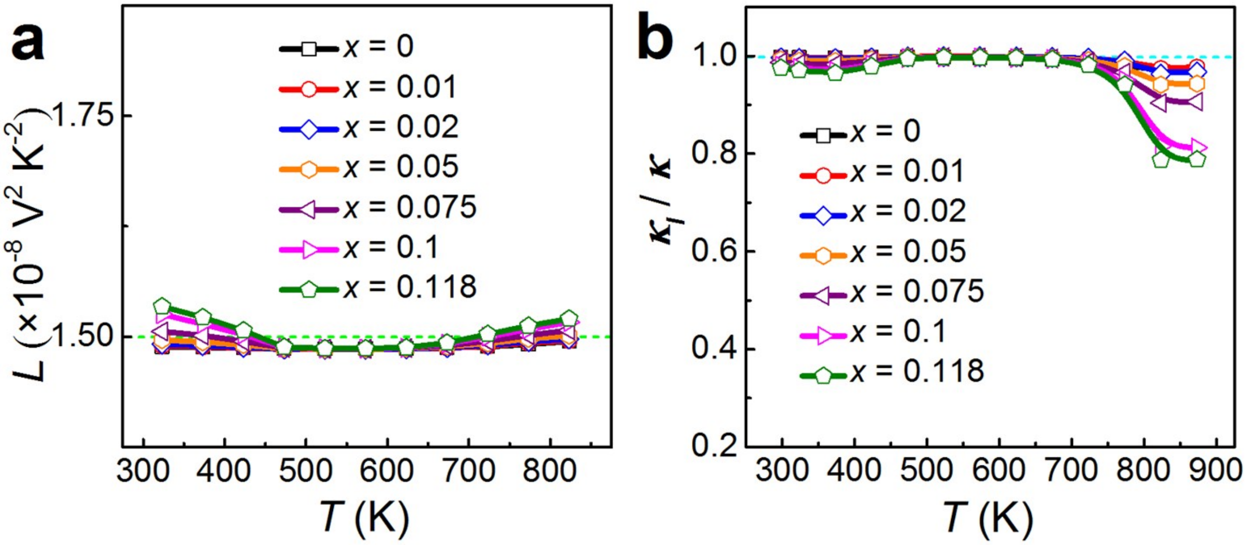

Figure S6. Plots of (a) calculated *T*-dependent *L* and (b) calculated *T*-dependent κ_l/κ ratio.

Section 7. Comparison of *ZT*.

Figure S7 provides the comparison of both average and peak *ZT* with reported Cu-doped works from 298 K to 873 K.[6, 7] Our heavily Cu-doped SnSe achieves a high peak *ZT* of 1.41 at 823 K and competitive average *ZT* of 0.35 for the entire temperature range, which is very competitive compared with reported Cu-doped SnSe studies.

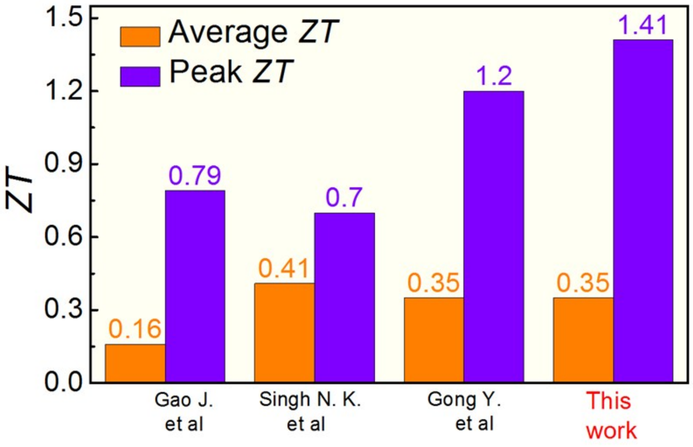

Figure S7. The comparison of both average and peak *ZT* with reported Cu-doped SnSe works from 298 K to 873 K.[6, 7]

To compare the *ZT* values in more detail, Tables S1 summaries both average and peak *ZT* with the similar studies of *p*-type doped SnSe. As can been seen, based on our high peak *ZT* of 1.41 at 823 K and competitive average *ZT* of 0.35 for the entire temperature range, our heavily Cu-doped SnSe is competitive compared with reported *p*-type doped SnSe.

Table S1. A comprehensive summary on the *ZT* of the *p*-type doped polycrystalline SnSe. Here solvothermal is abbreviated as ST, hydrothermal is abbreviated as HT, melting is abbreviated as M, zone-melting is abbreviated as ZM, annealing is abbreviated as A, solid-state solution is abbreviated as SSR, mechanical alloying is abbreviated as MA, and hot-pressing is abbreviated as HP. The average *ZT* means the average *ZT* values are calculated for the entire temperature range.

Product	Synthetic Method	Peak *ZT*	*T* (K)	Average *ZT*	Ref.
**11.8% Cu-doped SnSe**	**ST+SPS**	**1.41**	**823**	**~0.35**	**This work**
$Sn_{0.97}Cu_{0.03}Se$	M+HP	0.79	823	~0.16	6
$Sn_{0.98}Cu_{0.02}Se$	M+A+SPS	0.7	773	~0.41	7
$Sn_{0.99}Cu_{0.01}Se$	HT+SPS	1.2	873	~0.35	8
$Sn_{0.99}Ag_{0.01}Se$	M+A+HP	0.6	750	~0.27	9
$Sn_{0.99}Ag_{0.01}Se$	M+A+SPS	0.74	823	~0.31	10
$Sn_{0.985}Ag_{0.015}Se$	M	1.3	773	~0.49	11
$Sn_{0.97}Ag_{0.03}Se$	ST+SPS	0.8	850	~0.3	12
$Sn_{0.99}Na_{0.01}Se$	M+A+SPS	0.85	800	~0.32	13
$Sn_{0.99}Na_{0.01}Se$	M+SPS	0.75	823	~0.29	14
$Sn_{0.99}Na_{0.01}Se$	M+SPS	~0.8	800	~0.34	15
$Sn_{0.985}Na_{0.015}Se$	M+MA+HP	~0.8	773	~0.37	16
$Sn_{0.98}Na_{0.02}Se$	SPS	0.87	798	~0.35	17
$Sn_{0.97}Na_{0.03}Se$	SPS	0.82	773	0.44	18
$Sn_{0.99}Na_{0.005}K_{0.005}Se$	MA+SPS	1.2	773	~0.53	19
$Sn_{0.995}Na_{0.005}SeCl_{0.005}$	SSR+HP	0.84	810	~0.35	20
$Sn_{0.99}Na_{0.01}Se_{0.84}Te_{0.16}$	MA+SPS	0.72	773	~0.32	21
$(Sn_{0.96}Pb_{0.04})_{0.99}Na_{0.01}Se$	M+SPS	~1.2	773	~0.47	22
$Sn_{0.99}K_{0.01}Se$	MA+SPS	~1.1	773	~0.49	23
$Sn_{0.995}Tl_{0.005}Se$	M+HP	0.6	725	-	24
$Sn_{0.99}In_{0.01}Se$	M+HP	0.2	823	~0.09	25
$Sn_{0.9}Ge_{0.1}Se$	M	-	400	-	26
$Sn_{0.96}Ge_{0.04}Se$	ZM+HP	0.6	823	~0.17	27
$Sn_{0.99}Zn_{0.01}Se$	M+HP	0.96	873	~0.29	28
$Sn_{0.97}Sm_{0.03}Se$	M+HP	0.55	823	~0.15	29
$SnSe_{0.985}Cl_{0.015}$	M	1.1	773	~0.26	11
$SnSe_{0.9}Te_{0.1}$	ST+SPS	1.1	800	~0.6	30

Section 8. Examination of the reproducibility for obtained thermoelectric properties.

To evaluate the reproducibility of our thermoelectric properties, we tested 6 times for our 11.8 % Cu-doped SnSe pellets from room temperature to 873 K. Figure S8 shows the *T*-dependent properties (σ, S, $S^2\sigma$, κ, and ZT) with different cycles. All properties were measured along directions perpendicular to the sintering pressure. The 1st, 3rd and 5th measurements were taken under heating processes, and the other three measurements were taken under cooling processes. The results indicate that the reproducibility of our obtained thermoelectric properties is excellent under 873 K, and the phase transition at 800 K did not affect the stability of thermoelectric properties in our Cu-doped SnSe pellets to a great extent.

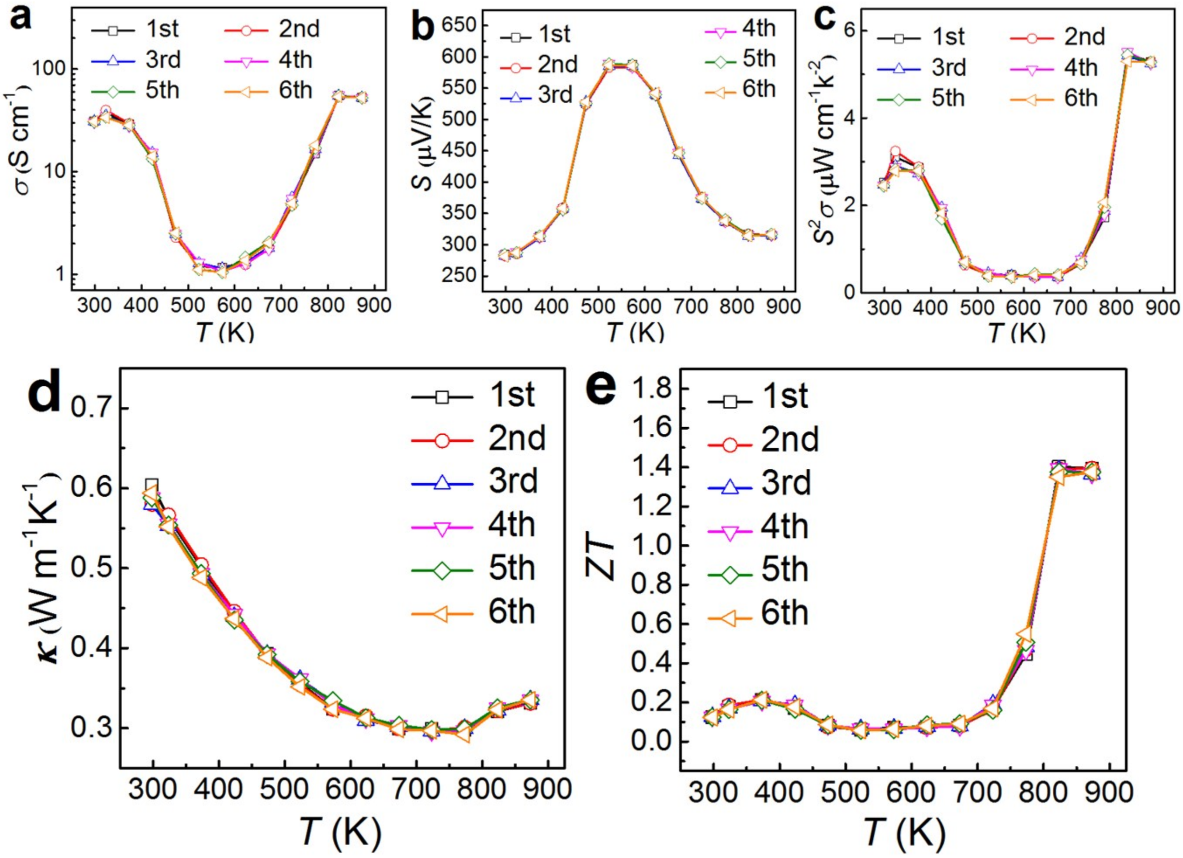

Figure S8. *T*-dependent properties with different measured times for our 11.8 % Cu-doped SnSe pellets: (a) σ; (b) S; (c) $S^2\sigma$; (d) κ; and (e) ZT. All properties are measured along ⊥ direction. The 1st, 3rd and 5th measurements were taken under heating processes, and the other three measurements were taken under cooling processes.

Reference

1. W. Kaminsky, *J Appl Crystallogr*, 2007, **40**, 382-385.
2. Y. Xu, W. Li, C. Wang, J. Li, Z. Chen, S. Lin, Y. Chen and Y. Pei, *Journal of Materials Chemistry A*, 2017, **5**, 19143-19150.
3. J. Shen, Z. Chen, S. lin, L. Zheng, W. Li and Y. Pei, *Journal of Materials Chemistry C*, 2016, **4**, 209-214.
4. X. She, X. Su, H. Du, T. Liang, G. Zheng, Y. Yan, R. Akram, C. Uher and X. Tang, *Journal of Materials Chemistry C*, 2015, **3**, 12116-12122.
5. L. Zhao, S. M. K. N. Islam, J. Wang, D. L. Cortie, X. Wang, Z. Cheng, J. Wang, N. Ye, S. Dou, X. Shi, L. Chen, G. J. Snyder and X. Wang, *Nano Energy*, 2017, **41**, 164-171.
6. J. Gao and G. Xu, *Intermetallics*, 2017, **89**, 40-45.
7. N. K. Singh, S. Bathula, B. Gahtori, K. Tyagi, D. Haranath and A. Dhar, *J Alloy Compd*, 2016, **668**, 152-158.
8. Y. Gong, C. Chang, W. Wei, J. Liu, W. Xiong, S. Chai, D. Li, J. Zhang and G. Tang, *Scripta Mater*, 2018, **147**, 74-78.
9. C.-L. Chen, H. Wang, Y.-Y. Chen, T. Day and G. J. Snyder, *J Mater Chem A*, 2014, **2**, 11171.
10. H. Leng, M. Zhou, J. Zhao, Y. Han and L. Li, *J Electron Mater*, 2015, **45**, 527-534.
11. L. Zhang, J. Wang, Q. Sun, P. Qin, Z. Cheng, Z. Ge, Z. Li and S. Dou, *Adv. Energy Mater.*, 2017, **7**, 1700573.
12. C.-H. Chien, C.-C. Chang, C.-L. Chen, C.-M. Tseng, Y.-R. Wu, M.-K. Wu, C.-H. Lee and Y.-Y. Chen, *RSC Adv*, 2017, **7**, 34300-34306.
13. T.-R. Wei, G. Tan, C.-F. Wu, C. Chang, L.-D. Zhao, J.-F. Li, G. J. Snyder and M. G. Kanatzidis, *Appl Phys Lett*, 2017, **110**, 053901.
14. H.-Q. Leng, M. Zhou, J. Zhao, Y.-M. Han and L.-F. Li, *RSC Adv*, 2016, **6**, 9112-9116.
15. T. R. Wei, G. Tan, X. Zhang, C. F. Wu, J. F. Li, V. P. Dravid, G. J. Snyder and M. G. Kanatzidis, *J Am Chem Soc*, 2016, **138**, 8875-8882.
16. E. K. Chere, Q. Zhang, K. Dahal, F. Cao, J. Mao and Z. Ren, *J Mater Chem A*, 2016, **4**, 1848-1854.
17. B. Cai, J. Li, H. Sun, P. Zhao, F. Yu, L. Zhang, D. Yu, Y. Tian and B. Xu, *J Alloy Compd*, 2017, **727**, 1014-1019.
18. K. Peng, H. Wu, Y. Yan, L. Guo, G. Wang, X. Lu and X. Zhou, *J Mater Chem A*, 2017, **5**, 14053-14060.
19. Z.-H. Ge, D. Song, X. Chong, F. Zheng, L. Jin, X. Qian, L. Zheng, R. E. Dunin-Borkowski, P. Qin and J. Feng, *J Am Chem Soc*, 2017, **139**, 9714-9720.
20. S. D. Yang, R. K. Nutor, Z. J. Chen, H. Zheng, H. F. Wu and J. X. Si, *J Electron Mater*, 2017, **46**, 6662-6668.
21. T. R. Wei, C. F. Wu, X. Zhang, Q. Tan, L. Sun, Y. Pan and J. F. Li, *Phys Chem Chem Phys*, 2015, **17**, 30102-30109.
22. Y. K. Lee, K. Ahn, J. Cha, C. Zhou, H. S. Kim, G. Choi, S. I. Chae, J.-H. Park, S.-P. Cho, S. H. Park, Y.-E. Sung, W. B. Lee, T. Hyeon and I. Chung, *J Am Chem Soc*, 2017, **139**, 10887-10896.
23. Y.-X. Chen, Z.-H. Ge, M. Yin, D. Feng, X.-Q. Huang, W. Zhao and J. He, *Adv Funct Mater*, 2016, **26**, 6836-6845.
24. V. Kucek, T. Plechacek, P. Janicek, P. Ruleova, L. Benes, J. Navratil and C. Drasar, *J Electron Mater*, 2016, **45**, 2943-2949.
25. J. H. Kim, S. Oh, Y. M. Kim, H. S. So, H. Lee, J.-S. Rhyee, S.-D. Park and S.-J. Kim, *J Alloy Compd*, 2016, **682**, 785-790.

26. M. Gharsallah, F. Serrano-Sanchez, N. M. Nemes, F. J. Mompean, J. L. Martinez, M. T. Fernandez-Diaz, F. Elhalouani and J. A. Alonso, *Sci Rep*, 2016, **6**, 26774.
27. Y. Fu, J. Xu, G.-Q. Liu, X. Tan, Z. Liu, X. Wang, H. Shao, H. Jiang, B. Liang and J. Jiang, *J Electron Mater*, 2017, **46**, 3182-3186.
28. J. C. Li, D. Li, X. Y. Qin and J. Zhang, *Scripta Mater*, 2017, **126**, 6-10.
29. J. Gao, H. Zhu, T. Mao, L. Zhang, J. Di and G. Xu, *Mater Res Bull*, 2017, **93**, 366-372.
30. M. Hong, Z.-G. Chen, L. Yang, T. C. Chasapis, S. D. Kang, Y. Zou, G. J. Auchterlonie, M. G. Kanatzidis, G. J. Snyder and J. Zou, *J Mater Chem A*, 2017, **5**, 10713-10721.
